# Cre/*lox*-Recombinase-Mediated Cassette Exchange for Reversible Site-Specific Genomic Targeting of the Disease Vector, *Aedes aegypti*

**DOI:** 10.1038/srep43883

**Published:** 2017-03-07

**Authors:** Irina Häcker, Robert A. Harrell II, Gerrit Eichner, Kristina L. Pilitt, David A. O’Brochta, Alfred M. Handler, Marc F. Schetelig

**Affiliations:** 1 Institute for Insect Biotechnology, Justus-Liebig-University Giessen, Heinrich-Buff-Ring 26-32, 35392 Giessen, Germany; 2 Institute for Bioscience and Biotechnology Research, University of Maryland, College Park, Rockville, Maryland 20850, USA; 3 Insect Transformation Facility, University of Maryland, College Park, Rockville, Maryland 20850, USA; 4 Mathematical Institute, Justus-Liebig-University Giessen, Arndtstrasse 2, 35392 Giessen, Germany; 5 Department of Entomology, University of Maryland, College Park, Rockville, Maryland 20850, USA; 6 USDA/ARS, Center for Medical, Agricultural and Veterinary Entomology, 1700 SW 23rd Drive, Gainesville, FL 32608, USA

## Abstract

Site-specific genome modification (SSM) is an important tool for mosquito functional genomics and comparative gene expression studies, which contribute to a better understanding of mosquito biology and are thus a key to finding new strategies to eliminate vector-borne diseases. Moreover, it allows for the creation of advanced transgenic strains for vector control programs. SSM circumvents the drawbacks of transposon-mediated transgenesis, where random transgene integration into the host genome results in insertional mutagenesis and variable position effects. We applied the Cre/*lox* recombinase-mediated cassette exchange (RMCE) system to *Aedes aegypti*, the vector of dengue, chikungunya, and Zika viruses. In this context we created four target site lines for RMCE and evaluated their fitness costs. Cre-RMCE is functional in a two-step mechanism and with good efficiency in *Ae. aegypti.* The advantages of Cre-RMCE over existing site-specific modification systems for *Ae. aegypti,* phiC31-RMCE and CRISPR, originate in the preservation of the recombination sites, which 1) allows successive modifications and rapid expansion or adaptation of existing systems by repeated targeting of the same site; and 2) provides reversibility, thus allowing the excision of undesired sequences. Thereby, Cre-RMCE complements existing genomic modification tools, adding flexibility and versatility to vector genome targeting.

Mosquitoes are vectors for many different parasites and viruses causing diseases such as malaria, dengue, yellow fever, and chikungunya among others. With the spread of vector species into new habitats due to global travel and transport, several diseases have rapidly spread into many countries in recent years, posing an increasing thread for human health. One approach towards eliminating vector-borne diseases is to target the vector itself. However, current strategies for vector control like elimination of breeding sites and the use of insecticides have serious limitations. Therefore, the demand for effective methods to control mosquito populations has become more and more pressing.

Several new approaches currently being investigated rely on transgenic technologies, such as the improvement of the sterile insect technique (SIT), which is based on the mass release of sterile male mosquitoes for population suppression by infertile matings with females in the field. Another potential strategy is to introgress transgenes conferring resistance to parasite or virus infection into populations of mosquitoes. In both cases, mosquito strains with new, specific traits are needed, and transgenic technologies allow tailoring strains specifically for each application. Transgenic methods are also key to functional genomics and comparative gene expression studies. Such studies are essential for a better understanding of vector biology and gene function and are the basis for the development of new transgenic systems for applications. Moreover, once developed, different transgenic systems need to be compared for their efficacy in the same genomic context.

Transposon-mediated germline transformation has been the state of the art method for the development of genetically modified insects for the past two decades, and is applicable to many different insect species, including mosquitoes. A major drawback, however, is the random integration of the vector construct into the genome. This can lead to gene disruption by insertional mutagenesis, which can be lethal or lead to decreased fitness of the transgenic lines. Moreover, variable position effects in the genome caused by cis-acting regulatory elements or chromatin configuration influence transgene expression, and can result in suppression or inappropriate expression of the transgene. This makes creation of optimal transgenic lines for application inefficient and is a major difficulty for functional studies of comparative gene expression. Thus, it would be desirable to identify optimal genomic target sites with respect to host strain fitness and transgene expression and then repeatedly target these sites for further modifications and to directly compare different transgenic systems.

One possible strategy for site-specific targeting of the genome is the use of site-specific recombination systems. These systems typically consist of a single recombination sequence integrated at a defined target (or landing) site in the genome, and an identical sequence in a donor vector containing the transgene construct of interest. Recombination between the two sites is facilitated by a system-specific recombinase that is injected with the donor vector into target site strain embryos. Systems commonly used, in mammals as well as insects, are the FLP/*FRT* recombinase system from the two-micron plasmid of *Saccharomyces cerevisiae*^1^, the Cre/*loxP* system from *Escherichia coli* phage P1^2^, as well as the phiC31/*att* system from *Streptomyces* phage phiC31^3^. Of these, only the integrase phiC31 has been used for site-specific single recombination in non-drosophilid genomes, including two *Aedes* species^4^,^5^,^6^, *Anopheles gambiae*^7^,^8^ and the Mediterranean fruit fly, *Ceratitis capitata*^9^. While advantageous for genomic targeting, these single recombinations between donor vector and genomic target site result in the integration of the complete donor plasmid, including resistance genes and regulatory elements within the vector backbone that may be unnecessary or unwanted. A more versatile targeting strategy that only allows genomic integration of a defined sequence from the donor vector, is the recombinase-mediated cassette exchange (RMCE) system^10^. RMCE allows double-recombination between two pairs of heterospecific recombination sites, such as *lox* or *FRT*, flanking DNA cassettes in the donor vector and target site. *Lox* and *FRT* sites consist of an asymmetric variable 8 bp core flanked by palindromic 13 bp sequences. For both, only homospecific sites having an identical core can recombine with one another, while heterospecific sites are incompatible. Thus, recombination between the homospecific sites in the genomic target site and the donor plasmid results in the exchange of the sequence cassette located between the heterospecific sites (see ref. 11). Since the target heterospecific sites are restored after recombination, RMCE allows repeated manipulations at the target site. FLP/*FRT* and Cre/*lox*-based RMCE in insects was first demonstrated in *Drosophila melanogaster*^11^,^12^,^13^, but only Cre/*lox*-RMCE was successfully applied to a non-drosophilid species thus far, the tephritid *Anastrepha suspensa*^14^. The major difference in the mechanism for phiC31-integrase recombination is that the recombination sites are not preserved. In contrast to Cre- or FLP-systems, *attP* sites and *attB* sites recombine to create incompatible *attL* or *attR* sites, thus preventing successive modifications (deletions or additions) to the target site. However, they still can be used for one-time, unidirectional RMCE by flanking the target and donor DNA cassettes with inverse *attP* and *attB* sites, respectively. This was demonstrated in *D. melanogaster*^15^, *Bombyx mori*^16^, and in *Ae. aegypti*^17^. In a different approach, donor vectors and target sites each having a combination of single *attP/B and FRT* or *loxP* sites have allowed a two-step iRMCE process. In this approach, the complete donor vector is first integrated by phiC31-based single recombination, thereby creating pairs of homospecific *FRT* or *loxP* sites, which then can recombine in a second step resulting in excision of the DNA cassette between the homospecific sites^17^. This two-step cassette-exchange system is a significant advance for mosquitoes, relative to entire plasmid integrations by single-site recombinations, but is also irreversible and incompatible for successive manipulations with the same recombination systems.

Thus the full potential for reversible and repetitive RMCE, using heterospecific recombination sites, has yet to be accomplished in *Aedes*, which we now explore in this report. Moreover, a critical aspect of any target site system is that the genomic target site lines exhibit fitness and viability parameters nearly identical to their non-modified wild type species. In addition, transgene expression should be minimally, if at all, affected by genomic position effects. As an initial step to create a series of optimal target site lines for *Ae. aegypti*, we describe the fitness costs of the different integration sites to identify the most fit lines for downstream modifications in addition to testing fitness following targeted RMCE at a landing site.

## Results

### Creation of landing site lines for RMCE in *Ae. aegypti*

The *Ae. aegypti* Higgs White Eye (HWE) strain was injected with the plasmid pXL-BACII_FRT_3xP3DsRed_FRT3_loxN-PUbAmCyan-lox2272 (AH452), providing one marker cassette for *FRT*-*FRT3* RMCE and one for *loxN-lox2272* RMCE. Of 623 injected embryos, 143 survived to adulthood (23% survival, Table 1). Adult survivors of each sex were grouped to form G_0_ families and backcrossed to HWE, resulting in 5 male pools and 3 female pools. Screening the G_1_ offspring for the presence of fluorescent markers resulted in 4 independent lines, AH0212-M1, AH0212-M2, AH0212-F1, and AH0212-F2, as confirmed by inverse PCR/Splinkerette analysis of the integration site (Supplementary Table S1). Integrations occurred in intergenic regions for AH0212-M1 and AH0212-F1, in the second exon of a possible integral component of membrane (AAEL006862) in line AH0212-F2, and in an intron of AAEL009520 in AH0212-M2.

Transgenic individuals of all lines only expressed the 3xP3DsRed marker and not the *D. melanogaster* polyubiquitin (*Dmel*PUb)-regulated AmCyan marker gene (data not shown) suggesting that the *Dmel*PUb promoter is not functional in *Ae. aegypti*. The lines were stably maintained in the laboratory for more than 12 generations and were inbred to homozygosity for more than five generations without noticeable fitness effects, except for AH0212-M1. This line had larval development times extended by about one day, and decreased female fecundity after regular inbreeding during strain maintenance.

A fifth line (V3-M30) was created after injecting HWE embryos with the plasmid pB_*FRT*_3xP3DsRed_*FRT3*_*loxN*-3xP3-*FRT5*-AmCyan-*lox2272*-*loxP*-*attPrev* (V3). Individuals from this line were positive for both fluorescent protein markers. The integration was mapped to a non-annotated region of contig AAGE02017872.

### Establishment of Cre-RMCE

To perform *loxN-lox2272* RMCE experiments, the homozygous landing site lines AH0212-F1 and -M2 were injected with a donor plasmid carrying the *loxN*-3xP3AmCyan-*lox2272* (AH460) recombination cassette. Injection of 560 AH0212-F1 embryos (Table 1) resulted in 8 male pools and 8 female pools whose G_1_ progeny were screened for the expression of AmCyan and DsRed. In total, 6 families expressed both markers in the eyes: AH0312-M3, -M7, -F2, -F3, -F4, and -F7. Thus, 3xP3AmCyan integration occurred in more than one third of the pools. Similar injections of AH0212-M2 embryos resulted in only one G_0_ family producing progeny expressing DsRed and AmCyan in the eyes, AH0513-M8. Assuming that each of the positive pools contained a minimum of one individual with a successful recombination event, and that approximately 50% of the injected individuals were fertile, the estimated recombination frequency was 5.5% in the first experiment (AH0212-F1), and less than 1.5% in the second (AH0212-M2) (Table 1).

**Figure 1:**
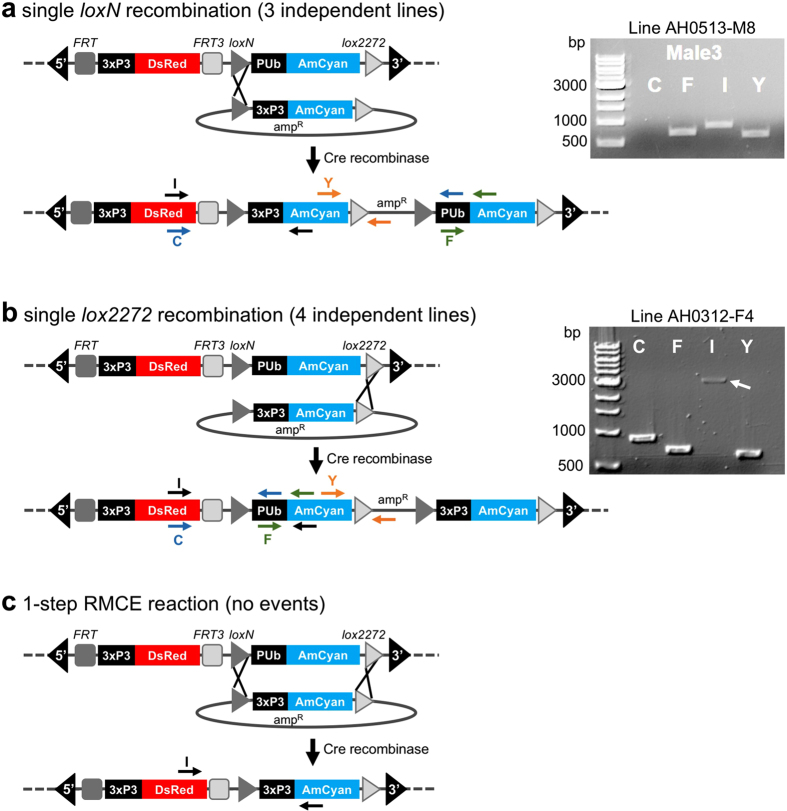
Recombination possibilities between *lox* sites of the RMCE landing site and the donor plasmid. Single recombination between either the *loxN* sites **(a)** or the *lox2272* sites **(b)** leads to the integration of the complete donor plasmid at the respective site. Arrows indicate PCR primer pairs that were used to identify which recombination event had taken place. Both *loxN* and *lox2272* single recombinations could be detected (3 and 4 independent lines, respectively). The combination of PCR products obtained for AH0513-M8 (A) indicate a *loxN* single recombination event instead of a cassette exchange, where only the I band would be expected. The PCR I band is 865 bp in case of *loxN* single recombination or an RMCE event. In case of a *lox2272* single recombination, the I band is 2.9 kb due to the larger size of the PUb promoter (2 kb vs 210 bp of the 3xP3 promoter), and the C band is present in addition (B). (**c**) Double recombination results in a 1-step cassette exchange (RMCE). This event was not observed in our experiments. 3′ and 5′ denote the *piggyBac* ends of the construct. 3xP3 is a universal eye-specific promoter, PUb is the *Dmel* polyubiquitin promoter. The DNA marker used for agarose gels was New England Biolabs 1 kb DNA ladder. bp = base pairs.

The red-and-cyan eye phenotype can arise by two mechanisms: donor plasmid integration via single recombination, or cassette exchange via double recombination (Fig. 1). To clarify the mechanism of recombination in the above lines, the landing sites were analyzed by PCR, which revealed that all lines arose from single recombination events at either the *loxN* or the *lox2272* site, resulting in the integration of the complete donor plasmid. This was indicated by either a combination of 710 bp (F), 865 bp (I), and 634 bp (Y) PCR products (i.e. a *loxN* integration event; Fig. 1a), or 887 bp (C), 710 bp (F), 2.9 kb (I) and 634 bp (Y) PCR products (i.e. a *lox2272* integration, Fig. 1b). None of the lines resulted in a single 865 bp (I) amplicon (Fig. 1c), which would indicate a cassette exchange. Finally, the presence of the target site marker gene PUbAmCyan (F band) was confirmed for all lines. Therefore, RMCE was not observed in this test, but rather donor plasmid integrations by single recombination reactions at either *lox* site that resulted in progeny having two pairs of homospecific *lox* sites in the same orientation.

To test if the wild type *loxP* site in combination with one of the mutated *lox* sites recombines more efficiently in *Ae. aegypti*, we performed *loxN-loxP* RMCE experiments. In two independent experiments, more than 1700 embryos of the landing site line V3-M30 were injected with the donor plasmid pSL_*loxN*-*3xP3EGFP-loxP* (Table 1). 175 adults arising from injected embryos were backcrossed in pools of up to 10 individuals, resulting in more than 18,000 G_1_ offspring that were screened for expression of the 3xP3-EGFP marker. However, EGFP fluorescence was not observed in any of the G_0_ families.

### *lox* integration lines further recombine to yield RMCE events at high efficiency

**Figure 2:**
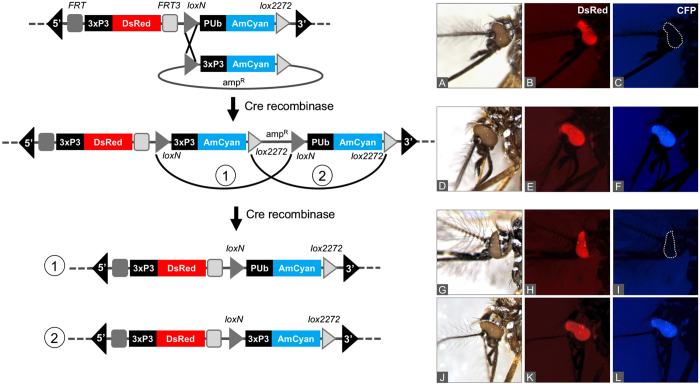
Two-step *lox*-RMCE in *Ae. aegypti* via a donor plasmid integration intermediate. Left: Schematic of a 2-step RMCE event; right: microscopic images showing the bright field image of the mosquito head (**A**, **D**, **G**, **J**) and the corresponding DsRed (**B**, **E**, **H**, **K**) and the CFP (**C**, **F**, **I**, **L**) fluorescence images. The landing site line displays only DsRed expression in the eyes as the *D. melanogaster* PUb promoter is not active in *Ae. aegypti* (**A**–**C**). A *loxN* single recombination event leads to donor plasmid integration, resulting in the additional expression of the AmCyan marker in the eyes (**D**–**F**). Embryonic micro-injection of this integration line with Cre recombinase results in recombination of either pair of homospecific *lox* sites. Recombination of the *loxN* sites (1) reverses the plasmid integration, resulting in a red-eyed phenotype (**G**–**I**), while recombination of the *lox2272* (2) sites completes the cassette exchange, resulting in a 2-step RMCE and unchanged phenotype (**J**–**L**).

**Figure 3:**
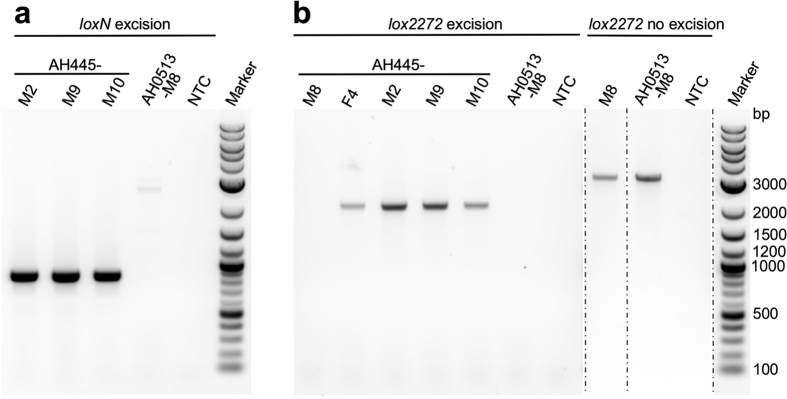
Agarose gel electrophoresis of control PCR products to confirm correct 3xP3-AmCyan excision via (**a**) *loxN* recombination or (**b**) PUb-AmCyan excision via *lox2272* recombination. AH445-F4, M2, -M8, -M9 and -M10 denote tested pools/families. AH0513-M8 is the parental line serving as a control for no excision events. (**a**) In case of correct *loxN* excision an 840 bp product is expected in the positive pools, but no product in the parental control due to a large product size (>5600 bp). (**b**) In case of correct *lox2272* excision a 2100 bp product is expected in the positive pools, but no product in the parental control (>8600 bp). To prove that a missing positive PCR band indeed was due to lack of excision, a no excision control PCR was done that would only produce a product if excision did not occur (shown here only for *lox2272*). See Supplementary Fig. S1 for the complete dataset. NTC represents the no template control reaction for the different primer combinations. Marker denotes the 2-log ladder from NEB. bp = base pairs.

Although double-recombination RMCE events were not evident in the G_1_ progeny screened, it was apparent that the presence and orientation of the resulting two pairs of homospecific *lox* sites could result in RMCE after a subsequent round of recombination. This would be similar to the incomplete phiC31-RMCE reactions in *Ae. aegypti* that were previously used as an intermediate for complete RMCE^17^. Either of the two homospecific *lox* pairs are predicted to recombine resulting in an excision event. While *loxN-loxN* recombination would result in excision of the 3xP3AmCyan marker, yielding offspring with only red eyes, and simply be a reversion of the original donor plasmid integration (Fig. 2), *lox2272-lox2272* recombination would excise the non-functional PUbAmCyan marker cassette and the vector backbone and thus result in RMCE. To test the possibility of completing RMCE, embryos of the *loxN* integration line AH0513-M8 were injected with Cre-recombinase plasmid (AH445). Screening of G_1_ progeny of 56 G_0_ adults grouped into 17 pools revealed 8 pools (AH445- F2, -F3, AH445-M1, -M2, -M5, -M8, M9, M10) in which the AmCyan marker was no longer visible, indicating that excision occurred involving the *loxN* sites (Table 1, Supplementary Table S1, Supplementary Table S2). PCR analysis (Fig. 3a) and sequencing of the PCR products from 3 of the 8 positive families confirmed *loxN* excision. The high frequency at which we observed this event indicated that in many of the pools a *lox2272* excision might have taken place as well. Therefore, 10 to 50 red-and blue-eyed larvae/pupae from each of the 17 pools were analyzed by PCR confirming excision of the *lox2272*-PUb-AmCyan-*lox2272* cassette in 6 pools, resulting in the desired RMCE product (Fig. 3b, Supplementary Fig. S1, and Supplementary Table S2). Thus, Cre-RMCE was achieved in *Ae. aegypti* in a two-step process that required independent reactions, with homospecific *lox* integration lines serving as an intermediate that reacted with high efficiency to complete the cassette exchange.

### Fitness tests of landing site and integration lines

To test the effect of transgene insertions at different genomic positions on the fitness of the AH0212 target site lines, several standard fitness parameters were investigated and compared to the reference line HWE. The integration line AH0513-M8 was also tested to determine if the *lox*-site mediated plasmid DNA integration in the AH0212-M2 landing site has any secondary effects on line fitness. Extensive statistical sample size estimation was performed prior to testing to ensure sufficient sensitivity for detecting differences of 10 percent points between the lines and HWE (Supplementary Table S4). For all tests a variation between replicates was observed, which was significant in most cases. This variance was neither consistent across tests for one line, nor across lines for one test, except for adult longevity, where the third replicate showed reduced male and female longevity across all lines (Supplementary Fig. S2). The reason for this is not clear and would need more in-depth analysis. Variance in other tests and replicates cannot be explained by experimental conditions and might indicate a possible interaction between replicate and line (i.e. an influence of the line on the effect of the replicate). The experimental setup was not designed to test for an interaction between replicate and line, as it was not the scope of this project. Nevertheless, it was included in the statistical analysis using a two-factorial mixed-effects ANOVA (treating replicate and the interaction between replicate and line as random effects), which reduced the overall significance between lines compared to an analysis not considering the interaction (Supplementary Table S3).

#### Fecundity

The numbers of eggs oviposited per female during an 18 hr overnight period were counted for three replicate experiments (85 to 108 females in total for each transgenic line and 218 for HWE; Supplementary Table S4). The mean number of eggs per female ranged from 93 for AH0212-M1 to 117 for AH0212-M2 (Table 2). It was significantly different between HWE and AH0212-M1 (p = < 0.001), HWE and AH0212-F1 (p = 0,005), as well as between AH0212-F1 and -M2 (p = 0.0016), AH0212-M1 and -M2 (p = < 0.001), and AH0212-M1 and –M8 (p = 0.0414) (Table 2, Supplementary Fig. S3).

#### Fertility

The hatch rates of eggs per female were compared for different nutrient conditions (more than 2000 eggs in total per transgenic line and more than 4000 for HWE). When eggs were hatched in water with only yeast, most of the larvae took between 24 and 48 hrs to hatch, whereas submerging the eggs in water supplied with fish food resulted in fast hatching of the majority of larvae (>90%) within 24 hr (data not shown). Overall, hatch rates within one line differed strongly when comparing yeast to fish food for all lines including the HWE reference, ranging from 14% to 47% for yeast, and 64% to 78% for fish food. This difference was highly significant (Welch two sample t-test, p < 0.001 for all lines). Moreover, the variability of the hatch rates between repetitions within one line was stronger with yeast than with fish food (Table 2, Supplementary Fig. S2). Also differences in hatch rates of the transgenic lines compared to HWE, as well as between the lines, were much more pronounced under low nutrient conditions than with optimal nutrients. With yeast, we observed significant differences in hatch rates between HWE and AH0212-M2 (p < 0.001), HWE and AH0513-M8 (p < 0.021), between AH0212-M2 and –F1 (p < 0.014), and between AH0212-M2 and -M1 (p < 0.021). Differences showed a higher significance if the interaction between replicate and line was not considered, and additional significant differences between HWE and AH0212-F1 and –M1, and between AH0212-M2 and AH0513-M8 were observed (Supplementary Table S3). When hatched with fish food, differences between lines did not reach significance when the interaction between replicate and line was considered in the analysis (Supplementary Table S3, Supplementary Fig. S3). Thus, fitness costs due to the transgene construct integration were more pronounced under more restrictive conditions.

#### Larval development time

The larval development period from hatching to pupation was determined following three replicate experiments. A total of 850 to 950 larvae were monitored for each transgenic line, and approximately 2000 larvae for HWE. Mean pupation time ranged from 6.2 days for HWE to almost 7 days for AH0212-M1 (Table 2). Larval development times for AH0212-M1 and –M2 was significantly different from HWE (p < 0.001, and p = 0.031, respectively), and between AH0212-M1 and AH0212-F1 and AH0513-M8 (p = 0.015 and p = 0.047, respectively). The development time of AH0513-M8 and the parental line AH0212-M2 was very similar (Supplementary Fig. S3). Not considering the interaction between replicate and line, all pairwise comparisons were significantly different except for AH0212-M2 and AH0513-M8 (Supplementary Table S3).

#### Adult longevity

Mean adult longevity was tested under mating conditions with equal numbers of males and females per cage, but without blood feeding. 467 to 624 adults were counted in total for each transgenic line and 1083 for HWE. At the termination of the experiment on day 81 less than five females and 2 males were still alive across all cages of the three replicates. Adult male longevity was very similar for all lines (21.5 to 22 days) except for AH0212-M1, which had a markedly longer average survival time of 25.4 days (Table 2). However, due to the variance between replicates, this difference did not reach significance (Supplementary Fig. S3). Despite strong variation between replicates also in female longevity tests, the differences between lines were more pronounced for females (Table 2 and Supplementary Fig. S3). In contrast to male longevity AH0212-M1 females had the shortest average survival time (36.7 days), which was significantly different from HWE and AH0212-F1. Moreover, the mean survival time of HWE and AH0212-F1 was significantly longer than that of AH0212-M2 and AH0513-M8 (p < 0.001 for all pairwise comparisons). Similar to the larval development time, the longevity of AH0513-M8 and AH0212-M2 adults was almost identical.

#### Male mating competitiveness

Mating competitiveness of transgenic males was tested with the Exact binomial test (two-sided, conf. level = 0.95). As AH0212-M1 had been observed to show decreased fitness (female fecundity and larval development time) upon repeated rounds of inbreeding during standard strain maintenance, the competitiveness of AH0212-M1 was tested at two different inbreeding stages: 3rd generation of inbreeding after backcross to HWE, and 5th generation of inbreeding after backcrossing. In total, egg collections from 192 to 238 females per transgenic line were analyzed, and 52 for AH0212-M1 inbreeding stage III.

The null hypothesis of equal competitiveness was only rejected for AH0212-M1 inbred generation V (p < 0.001), but not for any of the other transgenic lines tested, nor for AH0212-M1 inbred stage III. However, a significant difference in competitiveness might have been missed for the latter due to the small sample size, for which no more than a 20% difference could have been detected. Thus, under the conditions tested all transgenic lines except AH0212-M1 inbred generation V were equally competitive to the HWE strain. No significant differences between replicates were observed (data not shown).

## Discussion

Genetic modification of the mosquito genome is an important tool to implement novel strategies to control vector insects or the pathogens they transmit. Site-specific genome modification methods allow targeting of genomic positions known to have none, or minimal impact on strain fitness and transgene expression, which is a prerequisite for the success of each transgenic strategy for vector control. Moreover, they allow the comparison of different transgene constructs unbiased by position effects, facilitating the identification of the most effective transgenes or systems for control applications and functional genomics studies to investigate gene function and relative expression levels. This cannot be easily achieved by transposon-mediated germline transformation due to the essentially random genomic integration of the transposon vector. Cre/*lox*-based RMCE has been used successfully in *D. melanogaster*^12^ and *A. suspensa*^14^, and would be a versatile tool for *Ae. aegypti* transgenesis, among other insect species. In contrast to phiC31-RMCE or iRMCE^17^, *lox*-RMCE would allow for repetitive target site modification by cassette replacement or deletion, thus facilitating strain development for downstream applications.

Here, we report Cre-RMCE experiments using two different combinations of heterospecific *lox* sites, *loxN-lox2272* and *loxN-loxP*. For a ‘one-step’ cassette exchange, recombination has to occur simultaneously or in rapid succession at both recombination sites flanking the DNA cassettes. Unlike previous reports of RMCE^11^,^12^,^15^,^16^,^17^, Cre-RMCE in *Ae. aegypti* was shown to be functional not as a one-step but a two-step process involving *loxN* or *lox2272* sites, for which the initial recombination product containing the entire RMCE donor plasmid serves as a reaction intermediate, similar to incomplete phiC31-RMCE in *Ae. aegypti*, where single recombination side-products may also undergo a second recombination following injection of integrase to yield the final RMCE product^17^. Recombinations with a *loxN-loxP* landing site line (V3) were not observed. Possibly, the *loxP* site or its combination with *loxN* works less efficient in *Ae. aegypti* than *lox2272* (in combination with *loxN*). Another reason might be the genomic position of the landing site in the V3 line. Accessibility to the site might be impaired due to chromatin conformation.

Single-site recombinations during RMCE experiments resulting in donor plasmid integrations were observed previously in different RMCE approaches depending on the targeted species. In *Drosophila*, single-site *lox* recombination events were detected in addition to double recombination Cre-RMCE events (frequencies not stated^12^), while single and double recombination occurred at nearly equivalent rates for FLP-RMCE^11^. In *A. suspensa*, only RMCE events were observed with the Cre/*lox* system, though these occurred at a low frequency from only 8 fertile G_0_ lines^14^. For phiC31-RMCE, only cassette exchange events were observed in *Bombyx mori*^16^, while additional single recombination events were observed in *D. melanogaster* and *Ae. aegypti*^15^,^17^.

The mechanism of RMCE is assumed either as a simultaneous double crossover or two individual crossover reactions in quick succession, as there was little evidence for stable and long-lived intermediates^18^. Considering the results presented here and the previous occurrence of single-site recombinants in Cre-, FLP, and phiC31-RMCE systems and the demonstration of their functionality as RMCE intermediates^17^, however, the formation of a stable intermediate reaction (i.e. integration) product might be a more common event in RMCE than previously assumed and points towards successive instead of simultaneous crossovers. Donor plasmid integration results in two pairs of equally oriented homospecific *lox* sites that could recombine in an excision reaction. In general, intramolecular excision is kinetically and thermodynamically favored over bi-molecular integration^10^,^19^,^20^. Therefore, single recombination products occurring in Cre- or FLP-RMCE experiments should be inherently unstable as long as more than a threshold level of recombinase activity is present^10^,^19^,^20^. Excision via the recombination integration sites would result in a simple reversion of the integration while excision via the other pair of homospecific sites would result in completion of RMCE. The relative efficiencies of the three processes (integration versus two different possibilities of excision) may affect the recovery rate of single-site or dual-site recombinants. We speculate that excision reactions might occur more immediately between the recombination integration sites than between the resulting other two homospecific sites, probably because recombinase protomers are still bound to these sites. Thereby, the integration is immediately reversed before the second intramolecular recombination could take place, leading to the low recovery rate of one-step RMCE events. This is supported by the finding that once individuals with stable single-site recombinant integrations are recovered, subsequent excisions may be generated straightforwardly in an independent experiment, where the two homospecific *lox* pairs react with similar efficiency to either yield reversion of integration or completion of two-step RMCE (Supplementary Table S2). Nevertheless, complete one-step RMCE events are obtained in *D. melanogaster* with an estimated frequency of 9% for Cre-RMCE, and up to 30% for FLP-RMCE^11^,^12^. Possibly, species-specific factors impede the integration reaction or further promote its reversion in *Ae. aegypti*, resulting in the low recovery of one-step RMCE events. Extensive investigations e.g of chromatin structure at or around the recombination target sites, additional protein factors involved or relative recombinase activities or binding affinities in *Ae. aegypti* compared to other insect species would be necessary to shed more light on this question. In the phiC31/*att* system, the recombination of *attP* with *attB* produces incompatible *attR* and *attL* sites. The reverse reaction of these sites cannot be catalyzed by phiC31 integrase. Thus, an integrative recombination is stable once it has occurred, which explains why one-step phiC31-RMCE functions with a better efficiency in *Ae. aegypti* than one-step Cre-RMCE.

Fitness tests were conducted under laboratory conditions and with small-scale populations. Of the four lines tested relative to the parental strain HWE, AH0212-M1 overall showed the highest fitness cost, including reduced female fecundity and fertility, prolonged larval development and reduced male competitiveness in the fifth homozygous inbreeding generation. Male competitiveness is a crucial prerequisite for an efficient SIT program. A reduction of male competitiveness was not observed in tests with the third AH0212-M1 inbreeding generation. However, small differences in competitiveness would have been missed due to the limited number of viable eggs that were available for this inbreeding stage, resulting in a sample size too small to detect differences smaller than 20%. The integration site of the transgene construct in AH0212-M1 is located in contig AAGE02015880.1 for which genes have not been annotated at or proximal to the landing site. Therefore, the basis for the fitness costs associated with this line is not clear. Regarding male competitiveness all other lines were equally competitive to HWE under the experimental conditions used and would be good candidates for further modification and testing. Further field cage mating experiments should be conducted beyond these lab-scale tests with even larger numbers but at lower densities to better reflect the field environment upon release. Moreover, for release purposes, the strains would need to be tested in comparison to wild type mosquitoes from the field.

Overall, AH0212-F1 is the most fit line among the lines tested, performing closest to the HWE reference strain in all tests except for female fecundity. The performance of AH0513-M8 was similar to the parental line AH0212-M2 in all tests. Thus, the additional integration of the 1.35 kb 3xP3-AmCyan construct at the AH0212-M2 landing site did not significantly impact the fitness of the line, although the landing site is within an intron. Integration of larger constructs, however, might interfere with proper transcript splicing and thus negatively impact line fitness. Similarly, integration of additional constructs at the landing site of AH0212-F2 (localized by annotation at an exon terminus) may be problematic. Taken together with the overall performance in the fitness tests, AH0212-F1 would therefore be the recommended landing site line for further genetic modifications.

The significant difference in fertility obtained with minimal and enriched nutrients indicates the strong potential effects of experimental conditions on the results of fitness tests. Consequently, data describing the fitness of transgenic lines obtained by different protocols are difficult to compare. Therefore, standardized guidelines for laboratory-scale (and eventually also field cage scale) fitness tests are required for each species of interest, in order to judge the fitness of transgenic lines created by different labs, and to have a useful resource of lines. Beyond these guidelines, protocols would have to be adapted to specific questions or applications, for example performance under mass rearing conditions.

## Conclusions

We have created and characterized four landing site lines for Cre-based RMCE genomic targeting and tested them for viability, fecundity, fertility, and male mating competitiveness to provide a resource of lines for genetic modifications and/or the study of gene function. Our results show that the Cre/*lox*-system is functional in *Ae. aegypti* and can be used for either site-specific integration or for RMCE in a two-step protocol. This is an important additional tool to the existing phiC31-RMCE and iRMCE targeting methods with the benefit of being reversible and subject to repetitive modification, thus allowing the excision of undesired sequences and the unlimited addition of new ones. Even when comparing Cre-RMCE to the versatile and site-specific CRISPR/Cas modification system, three advantages separate the technologies from each other. First, successive targeting of the same site, though in principle possible with CRISPR/Cas, is limited by the requirement for a PAM site, which might have been altered or destroyed during the previous modification. Secondly, CRISPR/Cas genome editing is not reversible. Third, our study could not find any off-target effects or pseudo integrations using the *loxN* and *lox2272* sites, suggesting an advantage over CRISPR/Cas-based systems where off-target effects have been reported in several species^21^,^22^,^23^,^24^,^25^, although this topic is constantly being addressed, e.g. by modifying the Cas nuclease^26^.

A one-step RMCE should be possible in theory, but most likely is a rare event due to the more favorable excision reaction, though this requires further study. Alternative approaches that could shift the equilibrium from excision to integration might offer a possibility to enhance one-step Cre-RMCE in *Ae. aegypti*, though nevertheless, the high efficiency of RMCE completion by a second-generation recombination is encouraging for the routine use of this versatile means of genomic targeting, and the two-step process herein provides more insights into the possible mechanism of a single-step RMCE.

## Methods

### Mosquito rearing

*Ae. aegypti* lines were reared in an insectary at constant conditions of 27 °C, 70% RH, and a 12:12 hr light:dark cycle, with larvae fed on Tetra TabiMin fish food pellets. Adult mosquito diet was 10% sucrose with adult females fed once a week with cow or pig blood that was purchased from a butcher shop.

### Germline transformation

Transgenic *Ae. aegypti* mosquitoes *for lox2272-loxN* or *FRT-FRT3* RMCE were created by injecting preblastoderm embryos (HWE) with the *piggyBac* vector, pXL-*BacII-FRT_3xP3DsRed_FRT3_loxN-PUbAmCyan-lox2272* (AH452), and a *piggyBac* transposase helper plasmid, *phsp-pBac*^27^ in embryonic injection buffer (EIB; 5 mM KCl, 0.1 mM NaPO_4_, pH 6.8) at a concentration of 150 ng/μl and 300 ng/μl, respectively. In addition, a plasmid mixture was included for monitoring the quality of injections consisting of a minimal *pMos* vector (pMos QC, 50 ng/μl) and a source of pMos transposase (pK*hsp82Mos*, 200 ng/μl)^28^.

Transgenic *Ae. aegypti* mosquitoes with additional recombination sites for RMCE were created by injecting HWE preblastoderm embryos with the *piggyBac* (pB) vector pB-*BacII-FRT_3xP3DsRed_FRT3_loxN-3xP3-FRT5-AmCyan-lox2272-loxP-attPrev* (V3) - and *phsp-pBac* in EIB at a concentration of 500 ng/μl and 200 ng/μl, respectively. To identify transgenic mosquitoes, *Ae. aegypti* L3 and L4 larvae or pupae were screened for fluorescent marker expression (see “Equipment and settings”).

### RMCE and excision experiments

*loxN-lox2272* RMCE experiments were performed by injecting preblastoderm embryos (lines AH0212-F1 and AH0212-M2) with 250 ng/μl of the donor vector, pSL_*loxN-3xP3AmCyan_lox2272* (AH460), and 150 ng/μl Cre-recombinase expressing plasmid (*phsp70-CRE*, (AH445)^14^ in EIB. *loxN-loxP* RMCE experiments were performed by injecting preblastoderm embryos (V3-M30 line) with pSL_*loxN-3xP3EGFP_loxP* (V20) and *phsp70-CRE* in EIB. The vector and Cre-expressing plasmid, respectively, were co-injected at the following concentrations: 250/250 ng/μl or 250/500 ng/μl.

Excision via *lox* site recombination was achieved by injection of AH0513-M8 preblastoderm embryos with *phsp70-CRE* at 200 ng/μl or 400 ng/μl in EIB.

### Equipment and settings

Screening for transgenic mosquito larvae or pupae was performed with a Leica M165 FC stereo microscope or a fully automated Leica M205FC stereo microscope with one of the following objectives: PLAN 0.8x LWD or PLANAPO 1.0x, using the following epifluorescence filters: DsRed (excitation 545/30 nm; barrier 620/60 nm), GFP-LP (Excitation 425/60 nm barrier 480 LP nm), YFP (excitation 510/20 nm; barrier 560/40 nm), CFP (excitation 436/20 nm; barrier 480/40 nm). Bright field and fluorescent image acquisition of adult mosquitoes was performed with a fully automated Leica M205FC stereo microscope with a PLANAPO 1.0x objective and a 1x Leica DFC7000 T camera. Image acquisition was performed at a 40–60-fold magnification, with an aperture of 1, binning of zero, gain of 1.5 to 3. The image acquisition software was Leica LAS X. Adobe Photoshop CS5.1 was used as image processing software to enhance screen display and print image (fluorescent signal over the background) of the adult mosquito pictures: moderate changes to image brightness and contrast was equally applied across the entire image, as well as moderate gamma changes.

### Restriction enzymes and primers

All restriction enzymes used were from New England Biolabs (NEB). All primer sequences are provided in Supplementary Table S5.

### Cloning

The vector pB-*BacII-FRT_3xP3DsRed_FRT3_loxN-3xP3-FRT5 AmCyan-lox2272-loxP-attPrev* (V3) was generated by recombining three fragments via Gibson Assembly kit (NEB) as follows: a 5.24 kb fragment obtained by an *Eco*RV-*Spe*I-double digest of AH452 (pXL-*BacII-FRT_3xP3DsRed_FRT3_loxN-PUbAmCyan-lox2272*) containing the *FRT_3xP3DsRed-SV40_FRT3* cassette, 2) a 360 bp PCR fragment of *loxN_3xP3_FRT* isolated with primer mfs47 and mfs48 from AH443^29^, and 3) a 1360 bp PCR fragment containing *AmCyan-SV40_lox2272_loxP_attPrev* isolated with primer mfs49 and mfs50 from AH443. Primer mfs48 contains base changes that mutate the *FRT* site of AH443 into an *FRT5* site. The Q5 High-Fidelity Polymerase (NEB) was used according to the manufacturer’s instructions.

The vector pXL-*BacII-FRT_3xP3DsRed_FRT3_loxN-PUbAmCyan-lox2272* (AH452) was generated by ligating the vector backbone of the *Bam*HI-*Bgl*II-double digested vector pXL*-BacII-3xP3-ECFP* (kindly provided by M. Fraser; see http://piggybac.bio.nd.edu) to the *FRT_3xP3DsRed_FRT3_loxN-PUbAmCyan-lox2272* cassette isolated by an *Fse*I cut of vector pSL_*FRT_3xP3DsRed_FRT3_loxN-PUbAmCyan-lox2272* (M1180). M1180 was created by ligating the PCR fragment *3xP3DsRed*, isolated using primer pair P964-P965 on pBac{*3xP3-DsRedaf* }^30^ to the *Afl*II cut vector pSL_*FRT_3xP3EGFP_FRT3_loxN-PUbAmCyan-lox2272*.

The vector pSL_*loxN-3xP3EGFP_loxP* (V20) was generated by recombining three fragments via Gibson Assembly kit (NEB) as follows: 1) a 3.95 kb fragment obtained by *Afl*II, *Kpn*I digest of M746 (p_*loxN-3xP3FRTAmCyan-lox2272-loxP-FRT*)^31^ containing *loxN-3xP3*, 2) a 815 bp PCR fragment of *EGFP-SV40* isolated with primer P76 and P77 from AH459, 3) a 250 bp PCR fragment containing *SV40-loxP* isolated with primer P78 and P148 from AH460. Primer P148 contains base changes that mutate the *lox2272* site of AH460 into a *loxP* site. The Q5 High-Fidelity Polymerase (NEB) was used according to the manufacturer’s instructions. The vector pSL_ *loxN_3xP3EGFP_lox2272* (AH459) was generated by ligating the PCR fragment *FRT_3xP3EGFP_FRT3*, isolated using primer pair P977-P978 on pMi_3xP3-EGFP (kindly provided by A. Klinakis, A. Babaratsas, and C. Savakis), to the *Eco*RI-*Nhe*I-digested pSLfa1180fa vector backbone^32^. The vector pSL_*loxN_3xP3AmCyan_lox2272* (AH460) was generated by cutting M879^14^ with *Bam*HI and performing a self-ligation.

### Genomic DNA extraction

Genomic DNA was extracted from individual mosquitoes according to Ashburner’s Protocol #48 method^33^ without the Phenol/Chloroform/Isoamylalcohol extraction or to the protocol of DNAzol Reagent (Life Technologies). DNA was resuspended in 50 μl ddH2O.

### Analysis of genomic landing sites

Genomic locations of the landing sites and their derived recombination lines was determined either by Genotyping Splinkerette PCR or by inverse (+nested) PCR according to one of the following protocols:

*Splinkerette PCR* was performed according to the protocol of Potter *et al*.^34^ with the modification of using 5.0 μl of gDNA in the enzymatic digestions. PCR products were extracted from 1.25% agarose gels using Qiaquick Gel Extraction kit (Qiagen). PCR products were either subcloned in vectors pCR-Blunt II TOPO or pCR4Blunt-TOPO (Life Technologies) or directly sequenced. DNA was extracted from transformants using Qiaspin Miniprep kit (Qiagen), and the correct insert size checked by *Eco*RI restriction digestion. Positive plasmids were sequenced using M13F and M13R-pUC primers flanking the inserts. Alternatively, PCR products were directly sequenced with nested primers anchored in the *piggyBac* terminal ends (5′SPLNK-PB-SEQ and 3′SPLNK-PB-SEQ). Resulting sequence reads were analyzed by comparison to the *Ae. aegypti* genome using BLAST at Vectorbase.org.

*Inverse PCR* was performed with *Hpa*II or *Hae*II restriction digestion of 190–330 ng of genomic DNA for 2.5 hrs at 37 °C in a volume of 30 μl with 50 U RNase I (NEB). Digested DNA fragments were self-ligated at a 10-fold dilution of the restriction reaction in 1x NEB buffer 1, 6.66 mM DTT, 0.33 mM ATP, 2 μl T4 DNA Ligase (NEB) at 4 °C overnight^35^. 9 μl of the self-ligated fragments were PCR amplified with primers newiPCR-5′PB and 157 R for the 5′pB end, and newiPCR-3′PB and 3′RevNew2 for the 3′pB end in 1x Phusion HF buffer, 200 nM of each primer, 200 μM dNTPs, 0.25 μl Phusion polymerase (Finnzymes) in a total volume of 25 μl. PCR cycling conditions for 5′pB end were: 3 min at 98 °C, 35 cycles of [30 s at 98 °C, 30 s at 58 °C, 2 min at 72 °C], 10 min at 72 °C. Annealing temperature for 3′pB end primers was 56 °C. Resulting PCR products were gel extracted (1.5% agarose) using the Qiaquick Gel Extraction kit, and PCR was directly sequenced and analyzed by BLAST as above.

*Semi-nested inverse PCR*. Genomic location of the landing site in line V3-M30 was determined by inverse and semi-nested PCR. Genomic DNA (700 ng) was *Msp*I-digested according to standard NEB protocols for 4 hr at 37 °C. Digested DNA was precipitated, self-ligated in 100 μl total volume containing 1x T4 Ligation Buffer and 2 μl T4 DNA ligase (NEB) overnight at 16 °C, precipitated and dissolved in 50 μl. 3 μl DNA was PCR amplified in 20 μl containing 1x Platinum Taq buffer, 1.5–2 mM MgCl_2_, 100 μM dNTPs, 500 nM of each primer, 0.2 μl Platinum Taq polymerase (Invitrogen). Cycling conditions for touchdown inverse PCR (iPCR) were: 90 s at 95 °C, 5 cycles of [30 s at 94 °C, 20 s at Tm + 5 °C, reduce by 2 °C per cycle, 2 min at 72 °C], and 30 cycles of [30 s at 94 °C, 20 s at Tm minus 5 °C, 2 min at 72 °C], 3 min at 72 °C (annealing temperature adjusted for each primer pair). The iPCR reaction was diluted 1:100, and 1 μl used for the semi-nested PCR. Cycling conditions for semi-nested PCR (nPCR) were: 95 °C for 90 s, 35x [94 °C for 30 s, Tm minus 5 °C for 20 s, 72 °C for 2 min], 72 °C for 3 min, 4 °C hold. Primers for probing the 3′ *piggyBac* integration site were mfs12/P139 (iPCR) and mfs34/P139 (nPCR), and for probing the 5′ *piggyBac* integration site mfs10/mfs11 (iPCR) and mfs10/mfs31 (nPCR).

### Determining the mechanism of *loxN-lox2272* recombination

Four separate standard PCR reactions were performed using different sets of primer pairs spanning the regions where Cre-mediated *lox* recombination was expected resulting in either an RMCE or integration event (Fig. 1).

Primer set C: DsRedFor-3, pUBRev + 3: 887 bp band; Primer set F: pUBFor-3, AmCyan Rev + 3: 710 bp band; Primer set I: DsRedFor-3, AmCyanRev + 3: either 865 bp band (single *loxN* recombination or double recombination (cassette exchange)), or 2.9 kb band, (single *lox2272* site recombination); Primer set Y: AmCyanFor2, pSLRev2: 634 bp band. PCR reactions contained 1x Promega’s GoTaq colorless Master Mix, 1 μM of each primer, 1.0 μl genomic DNA in a total volume of 25.0 μl. PCR cycling conditions were 95 °C for 3 min, 25x [95 °C for 30 s, 58 °C for 30 s, 72 °C for 1 min], 72 °C for 5 min, 4 °C hold.

PCR product bands were isolated from 0.8% agarose gels and gel extracted using Qiaquick GelExtraction kit. Amplicons were subcloned into the vector pCR4_TOPO-TA (Life Technologies), and the resulting transformants were plasmid DNA extracted using Qiaspin Miniprep kit, diagnostic digested with *Eco*RI to check for inserts, and positive clone plasmids were sequenced using flanking (M13F) and/or internal (AmCyan-For2) primers. Resulting sequence reads were analyzed by sequence alignment comparison to putative single site *lox2272* or *loxN* recombination sequence maps.

### PCR analysis of *lox* excision events (AH445 lines)

Correct excision of 3xP3AmCyan via *loxN* recombination (phenotypical loss of AmCyan marker expression) was confirmed by PCR using primers P49 and mfs45 to yield a 840 bp product. A no excision control PCR was done with primers mfs3 and mfs45 (430 bp product). Phenotypically constant individuals (parental red and cyan eye phenotype) were analyzed for excision of PUbAmCyan via *lox2272* recombination using primers P52 and mfs10 (2100 bp product). The no excision control PCR was performed with primers P186 and mfs10 (3200 bp product). For higher throughput, 10 pupae were pooled, up to 50 pupae were analyzed per family. PCR reactions (Platinum Taq polymerase, Invitrogen), TOPO-cloning and sequencing were performed as described above and annealing temperatures adjusted for each primer pair.

### Fitness and competitiveness tests

For testing the fitness and competitiveness of landing site lines and integration lines the following parameters were assessed: female fecundity, female fertility, larval to pupal development time, female and male adult longevity, and male mating competitiveness. Requisite sample sizes to detect at least a 10% difference between lines were estimated (see below). To account for possible biological artifacts, the tests were split into 3 repetitions. Numbers in the experiments described below represent one repetition. Sample sizes in the experiments were chosen larger than the estimated necessary sample sizes to account for possible losses during the tests. All lines were inbred and screened for homozygosity for 3 to 4 generations previous to the setup of experimental cohorts (except line AH0212-M1, for which the inbred second generation was tested in addition, and line AH0513-M8 was inbred for 9 generations). All raw data are provided in Supplementary Table S6.

#### Establishment of experimental cohorts for fitness and competitiveness tests

One to 5 month old eggs from the different homozygous transgenic lines and the HWE host strain were hatched in the afternoon in oxygen-depleted water with 0,2% (w/v) baker’s yeast. The following morning L1 larvae were placed in rearing trays at a density of 0.4 larvae per ml water with all larval trays equally fed during rearing. If larvae were raised in more than one tray per line, pupae were combined from all trays before sexing based on genitalia morphology. All experimental individuals were screened for homozygosity according to the intensity of the fluorescent eye marker. Adults were allowed to emerge and reach maturation (3 days post-eclosion) at equal densities before setting up crosses for the fitness and competition tests.

#### Fecundity

For each transgenic line 2 inbreeding cages were established with 40 mature females and 20 mature males. Five cages were established for the HWE strain with adults allowed to mate for 7 days before blood feeding. Females that did not feed were removed. Two days later, the remaining females were transferred to individual oviposition cages and allowed to oviposit on day 3 post-blood feeding (pBF) on wet oviposition paper. Females were removed on day 4 pBF and the eggs incubated for 48 hrs, dried and stored at 27 °C, 70% humidity for 5–30 days before hatching to determine fertility (see below). Repetition 3 was conducted only with 15 females of each transgenic line and 30 HWE females.

#### Fertility

Oviposition papers with eggs collected for the purposes of determining fecundity (above) were divided in half and the number of eggs on each half of the oviposition paper counted, creating two batches of eggs per female. To test for the influence of nutrient availability, the two batches of eggs from each female were hatched separately under two different conditions: 1) tap water plus 0.2% yeast served as the low nutrient condition; and 2) tap water with fish food as the rich nutrient condition. Hatched larvae were counted at 24 hr, 48 hr, and 7 days after submerging the eggs in the low or rich nutrient water and the hatch rate determined as ratio of hatched larvae to laid eggs for each individual female.

#### Larval to pupal development time

Eggs from 7–10 females of each transgenic line and of 13–15 females of HWE were hatched in a large tray (30 × 40 cm, 1 L water) with some crumbs of fish food. The next day, 3–4 trays with 100 L1 larvae each at a density of 0.25 larvae per ml water were established for all transgenic lines and 6–7- trays for HWE. All trays were fed equally throughout the experiment. The day of hatching was set as day 0 while 24 hrs later, on day 1, trays of larvae were set up. Pupae were collected and counted daily in the morning and evening until all larvae had either pupated or died. No distinction was made between dead and living pupae at the time of counting. Larval death (number and time) were not noted.

#### Adult longevity

Pupae collected on day 6 and 7 during the larval-development test were separated by sex and allowed to emerge as adults at equal densities. For each line, 2–3 cages (20 × 20 × 20 cm) with an equal number of male and female adults (30–40 each) were then established, and 4–5 cages for the HWE reference strain. Dead male and female adults were collected and counted every third day starting on day 3 after cages were setup and continued until day 81. Individuals still alive on day 81 were noted.

#### Mating competitiveness

The mating competitiveness of each transgenic line was measured by combining 30 transgenic and 30 HWE mature adult males and adding 40 mature HWE females 24 hr later. Males were allowed to compete for mates for 7 days before blood feeding. Two days pBF, fed females were transferred into individual oviposition cages and allowed to oviposit on day 3 pBF. Females were removed on day 4 pBF and the eggs were incubated for 48 hrs before drying. Eggs from each female were hatched 4 to 10 days later by submerging into tap water supplemented with fish food. Larvae were screened at the L2 or L3 larval stage for the presence or absence of the fluorescent eye marker.

### Statistical analysis

Statistical analysis was performed using the open source software R, version 3.3.1^36^ together with the add-on R packages lme4^37^ (for two-factorial mixed-effects ANOVA models), car^38^ (for q-q-plots for model diagnostics), pbkrtest^39^ (for testing fixed effects in mixed-effects models), parallel^36^ (to accelerate computations by parallel processing), RLRsim^40^ (for testing random effects in mixed-effects models), survival^41^ (only for drawing survival curves), and multcomp^42^ (for multiple comparisons procedures).

#### Sample size estimation for fitness and competitiveness tests

Sample sizes for fecundity tests were estimated for the Tukey method of all pairwise 2-sided comparisons^43^ (under the assumption that, due to their typical average size, clutch sizes are approximately normally distributed, which, by exploratory data analysis, turned out to be an acceptable assumption). The sample size formula and the required 95%-quantile of the relevant studentized range were obtained from Horn & Vollandt^43^, p. 126, tab. 8.2 and tab. 4, p. 270, respectively. We considered 50% of the (unknown) standard deviation of the clutch sizes as the smallest relevant absolute difference to be detected with a power of 80% on a family-wise significance level of 5%.

Sample sizes for hatch rates (fertility tests) were estimated for all pairwise comparisons by means of two-sided two-sample binomial tests with family-wise significance level of 5% and a power of 80% to detect a difference of at least 10 percent points in hatch rates of which one is at least 85% and the other at most 75%. This was done analogously to Tukey’s method (Horn & Vollandt^43^ p. 126, tab. 8.2 and tab. 4, p. 270) using a normal approximation for the binomial distribution.

Sample sizes for male competitiveness tests were estimated for the two-sided one-sample binomial test (with continuity correction) of the hypothesis of a transgenic proportion of 50% (=equal competitiveness) for a significance level of 5% with a power of 80% for the detection of a difference of at least 10 percent points from the 50%-proportion. See, e.g., Fleiss *et al*.^44^ p. 30 ff.

The sample sizes for larval-development and adult longevity experiments were estimated for Tukey’s method of all pairwise two-sided comparisons (Horn & Vollandt^43^, p. 126, tab. 8.2 and tab. 4, p. 270) because development or survival times were intended to be completely observed (i.e., uncensored) and were assumed (due to their typical length) to be approximately normally distributed (if necessary after a log-transformation), which was confirmed by exploratory data analysis. The family-wise significance level was set to 5% and the power to 80% to detect the smallest relevant absolute difference of 50% of the (unknown) standard deviation of the development (or survival) times.

Sample size estimates for each experiment and obtained sample numbers in each experiment are listed in Supplementary Table S4.

#### Analysis of fecundity and fertility

Fecundity, as measured by the number of eggs laid per individual, and fertility, as measured by the hatch rate of eggs from individual females were analyzed using a two-factorial mixed-effects ANOVA, considering the replicate and the interaction between replicate and line (i.e., the influence of the line on the effect of replicate) both as random effects, and the genetic background (transgenic or HWE) as fixed effect (fit by the method of maximum likelihood with function lmer() of the R-package lme4^37^). The shortfall in actually achieved sample sizes for female fecundity for three lines (Supplementary Table S4) reduced the actual power to (not less than) 70% (or, equivalently, increased the smallest detectable absolute difference to (not more than) 55% of the standard deviation). The moderate deviations from normality observed in the fecundity data were neglected because of the large sample sizes. Hatch rate data were arcsine-transformed (after a linear mapping of the proportions interval [0, 1] onto the interval [−1, 1]) to come closer to normality. Fixed effects were tested with both a parametric bootstrap (i.e., a simulation-based) method and a Kenward-Roger approximation method using functions PBmodcomp() and KRmodcomp(), respectively, of the R-package pbkrtest^39^. (We used both methods to assess the reliability of the results.) Random effects were tested with simulation-based restricted maximum likelihood (REML) ratio tests implemented in function exactRLRT() of the R-package RLRsim^40^.

Both for fecundity and fertility analyses, pairwise comparisons of lines were performed by Tukey’s two-sided multiple comparisons of all means using function glht() of the R-package multcomp^42^ (with p-values adjusted by a single-step method), and corresponding, equivalent simultaneous confidence intervals for all differences in means were computed by the function confint() of the R-package multcomp. (In addition, Westfall’s truncated closed stepwise procedure for Tukey’s two-sided multiple comparisons of all means – a more powerful extension of the single-step method – was applied to potentially increase the number of significant differences. Its results were visualized by a “compact letter display” produced by function cld() of R-package multcomp. No corresponding, equivalent simultaneous confidence intervals were computed because they do not exist for Westfall’s stepwise procedure.) The family-wise significance level was set to 5% and, correspondingly, the family-wise confidence level to 95%. Further, a line-wise comparison of the influence of the food conditions on the (transformed) hatch rate was analyzed using Welch’s two-sided two-sample modified t-test per line followed by adjustment of p-values according to Holm’s method.

#### Analysis of larval to pupal development time and adult longevity

Replicates were pooled to plot pupation and adult survival curves per line. Since no censoring occurred pupation and survival times could be analyzed by mixed-effects ANOVAs and post hoc tests as above to compare the mean pupation time or mean longevity between lines and the HWE control. Pupation and male longevity times were log-transformed to get closer to normality. Moderate deviations from normality of the observed pupation and longevity times were considered negligible because of the large sample sizes.

#### Analysis of male competitiveness

Male mating competitiveness data were analyzed using an exact two-sided one-sample binomial test (R-function binom.test()). In addition, differences between the replicates performed per line were analyzed with Pearson’s chi-square test for equality of proportions in independent samples using the R function prop.test(). P-values were adjusted for multiple comparisons according to Holm.

##### Technical remark

Deviation from the statistical analysis plan on which the sample size estimations were based did not corrupt the intended test power because on the one hand, in the case of fertility and of pupal development and adult survival times, we obtained (partially extremely) much larger sample sizes, and on the other hand we incorporated into all analyses a source of variation (the replicates) as random effects which was not accounted for in the sample size estimation.

## Additional Information

**How to cite this article:** Häcker, I. *et al*. Cre/*lox*-Recombinase-Mediated Cassette Exchange for Reversible Site-Specific Genomic Targeting of the Disease Vector, *Aedes aegypti. Sci. Rep.* **7**, 43883; doi: 10.1038/srep43883 (2017).

**Publisher's note:** Springer Nature remains neutral with regard to jurisdictional claims in published maps and institutional affiliations.

## Supplementary Material

Supplementary Information

Supplementary Table S1

Supplementary Table S6

## Figures and Tables

**Table 1:** Summary of *Ae. aegypti* transformation and RMCE experiments.

experiment	[c] Donor ng/μl	[c] Helper ng/μl	parental line	embryos	larvae	% hatch	adults	% survival	pool size	lines
RMCE landing site lines	150^1^	300^2^	HWE	623	182	29	143	23	15	AH0212-F1, -F2, -M1, -M2
RMCE landing site lines	500^3^	200^2^	HWE	263	73	28	64	24	1–15	V3-M30
*loxN*-*lox2272* RMCE	250^4^	150^5^	AH0212-F1	560	263	47	218	39	15	AH0312-F2, -F3, -F4, -F7, -M3, -M7
*loxN*-*lox2272* RMCE	250^4^	150^5^	AH0212-M2	643	166	26	141	22	15	AH0513-M8
*loxN-loxP* RMCE	250^6^	150/500^5^	V3-M30	460	92	20	63	14	1–7	none
*loxN-loxP* RMCE	250^6^	250/500^5^	V3-M30	627/541	80/70	13	65/50	10	1–10	none
*lox* excision	0	200/400^5^	AH0513-M8	729/347	62/32	8,7	36/20	5	1–7	AH445-F2, -F3, -F4, -M1, -M2, -M5, -M8, -M9, -M10

^1^AH452, ^2^pBac helper, ^3^V3, ^4^AH460, ^5^phsp-Cre (AH445), ^6^V20.

**Table 2:** Fitness test results.

line	AH0212-F1	AH0212-M1	AH0212-M2	AH0513-M8	HWE
mean no. eggs	95.67^a,b^ ± 38.11	93.32^a^ ± 37.69	117.57^c^ ± 39.51	110.01^b,c^ ± 48.19	114.63^c^ ± 37.97
mean hatch rate ff (%)	76.2^a^ ± 21.81	71.92^a^ ± 21.43	72.86^a^ ± 24.39	64.47^a^ ± 27.1	78.65^a^ ± 18.06
mean hatch rate y (%)	37.55^b,c^ ± 27.38	34.82^b,c^ ± 26.44	14.39^a^ ± 16.26	26.38^a,b^ ± 21.39	47.5^c^ ± 28.22
mean pupation time (days)	6.41^a,b^ ± 0.65	6.94^c^ ± 0.8	6.45^b,c^ ± 0.56	6.52^a,b^ ± 0.65	6.22^a^ ± 0.48
rmean male survival (days)	21.9^a^ ± 0.714	25.4^a^ ± 0.77	21.5^a^ ± 0.707	21.7^a^ ± 0.928	22.0^a^ ± 0.55
rmean female survival (days)	45.4^b^ ± 0.975	36.7^a^ ± 0.864	40.2^a^ ± 0.983	39.0^a^ ± 1.043	45.2^b^ ± 0.679

Fitness test results for fecundity, fertility, larval development time and adult survival rate, showing mean or restricted mean with upper limit (rmean) and standard deviation (SD) or standard error of means (SEM), respectively. Means followed by the same letters within each row are not significantly different at the 0.05 level. ff = fish food, y = yeast.

## References

[b1] Andrews B. J., Beatty L. G. & Sadowski P. D. Site-specific recombination of the yeast plasmid two-micron circle: intermediates in the binding process. Basic Life Sciences 40, 407–424 (1986). 3551918 10.1007/978-1-4684-5251-8_31

[b2] Siegal M. L. & Hartl D. L. Transgene coplacement and high efficiency site-specific recombination with the Cre/*loxP* system in *Drosophila*. Genetics 144, 715–726 (1996). 8889532 10.1093/genetics/144.2.715 PMC1207562

[b3] Thorpe H. M. & Smith M. C. *In vitro* site-specific integration of bacteriophage DNA catalyzed by a recombinase of the resolvase/invertase family. Proceedings of the National Academy of Sciences, USA 95, 5505–5510 (1998). 10.1073/pnas.95.10.5505 PMC20407 9576912

[b4] Nimmo D. D., Alphey L., Meredith J. M. & Eggleston P. High efficiency site-specific genetic engineering of the mosquito genome. Insect Molecular Biology 15, 129–136 (2006). 16640723 10.1111/j.1365-2583.2006.00615.x PMC1602059

[b5] Franz A. W. . Comparison of transgene expression in Aedes aegypti generated by mariner Mos1 transposition and PhiC31 site-directed recombination. Insect Mol Biol 20, 587–598, doi: 10.1111/j.1365-2583.2011.01089.x (2011). 21699593 PMC3556457

[b6] Labbe G. M., Nimmo D. D. & Alphey L. *piggybac*- and PhiC31-mediated genetic transformation of the Asian tiger mosquito, *Aedes albopictus* (Skuse). PLoS neglected tropical diseases 4, e788, doi: 10.1371/journal.pntd.0000788 (2010). 20808959 PMC2923142

[b7] Meredith J. M., Underhill A., McArthur C. C. & Eggleston P. Next-generation site-directed transgenesis in the malaria vector mosquito *Anopheles gambiae*: self-docking strains expressing germline-specific phiC31 integrase. PLoS One 8, e59264, doi: 10.1371/journal.pone.0059264 (2013). 23516619 PMC3596282

[b8] Meredith J. M. . Site-specific integration and expression of an anti-malarial gene in transgenic Anopheles gambiae significantly reduces Plasmodium infections. PLoS One 6, e14587, doi: 10.1371/journal.pone.0014587 (2011). 21283619 PMC3026776

[b9] Schetelig M. F. . Site-specific recombination for the modification of transgenic strains of the Mediterranean fruit fly *Ceratitis capitata*. Proc Natl Acad Sci USA 106, 18171–18176, doi: 10.1073/pnas.0907264106 (2009). 19828439 PMC2775304

[b10] Baer A. & Bode J. Coping with kinetic and thermodynamic barriers: RMCE, an efficient strategy for the targeted integration of transgenes. Current Opinion in Biotechnology 12, 473–480, doi: S0958-1669(00)00248-2 [pii] (2001). 11604323 10.1016/s0958-1669(00)00248-2

[b11] Horn C. & Handler A. M. Site-specific genomic targeting in *Drosophila*. Proceedings of the National Academy of Sciences, USA 102, 12483–12488 (2005). 10.1073/pnas.0504305102 PMC1194931 16116081

[b12] Oberstein A., Pare A., Kaplan L. & Small S. Site-specific transgenesis by Cre-mediated recombination in *Drosophila*. Nat Methods 2, 583–585, doi: nmeth775 [pii] 10.1038/nmeth775 (2005). 16094382 10.1038/nmeth775

[b13] Wimmer E. A. Insect transgenesis by site-specific recombination. Nature Methods 2, 580–582 (2005). 16094381 10.1038/nmeth0805-580

[b14] Schetelig M. F. & Handler A. M. A Functional Comparison of the *3xP3* Promoter by Recombinase-Mediated Cassette Exchange in *Drosophila* and a Tephritid Fly, *Anastrepha suspensa*. G3 (Bethesda), doi: 10.1534/g3.112.005488 (2013). PMC3618355 23550127

[b15] Bateman J. R., Lee A. M. & Wu C. T. Site-specific transformation of Drosophila via phiC31 integrase-mediated cassette exchange. Genetics 173, 769–777, doi: 10.1534/genetics.106.056945 (2006). 16547094 PMC1526508

[b16] Long D. . *In vivo* site-specific integration of transgene in silkworm via PhiC31 integrase-mediated cassette exchange. Insect Biochem Mol Biol 43, 997–1008, doi: 10.1016/j.ibmb.2013.08.001 (2013). 23974010

[b17] Haghighat-Khah R. E. . Site-specific cassette exchange systems in the Aedes aegypti mosquito and the Plutella xylostella moth. PLoS One 10, e0121097, doi: 10.1371/journal.pone.0121097 (2015). 25830287 PMC4382291

[b18] Schlake T. & Bode J. Use of mutated FLP recognition target (*FRT*) sites for the exchange of expression cassettes at defined chromosomal loci. Biochemistry 33, 12746–12751 (1994). 7947678 10.1021/bi00209a003

[b19] Albert H., Dale E. C., Lee E. & Ow D. W. Site-specific integration of DNA into wild-type and mutant *lox* sites placed in the plant genome. Plant J 7, 649–659 (1995). 7742860 10.1046/j.1365-313x.1995.7040649.x

[b20] Araki K., Araki M. & Yamamura K. Targeted integration of DNA using mutant lox sites in embryonic stem cells. Nucleic Acids Res 25, 868–872 (1997). 9016639 10.1093/nar/25.4.868 PMC146486

[b21] Hsu P. D. . DNA targeting specificity of RNA-guided Cas9 nucleases. Nat Biotechnol 31, 827–832, doi: 10.1038/nbt.2647 (2013). 23873081 PMC3969858

[b22] Pattanayak V. . High-throughput profiling of off-target DNA cleavage reveals RNA-programmed Cas9 nuclease specificity. Nat Biotechnol 31, 839–843, doi: 10.1038/nbt.2673 (2013). 23934178 PMC3782611

[b23] Fu Y. . High-frequency off-target mutagenesis induced by CRISPR-Cas nucleases in human cells. Nat Biotechnol 31, 822–826, doi: 10.1038/nbt.2623 (2013). 23792628 PMC3773023

[b24] Cho S. W. . Analysis of off-target effects of CRISPR/Cas-derived RNA-guided endonucleases and nickases. Genome Res 24, 132–141, doi: 10.1101/gr.162339.113 (2014). 24253446 PMC3875854

[b25] Zhang X. H., Tee L. Y., Wang X. G., Huang Q. S. & Yang S. H. Off-target Effects in CRISPR/Cas9-mediated Genome Engineering. Mol Ther Nucleic Acids 4, e264, doi: 10.1038/mtna.2015.37 (2015). 26575098 PMC4877446

[b26] Kleinstiver B. P. . High-fidelity CRISPR-Cas9 nucleases with no detectable genome-wide off-target effects. Nature 529, 490–495, doi: 10.1038/nature16526 (2016). 26735016 PMC4851738

[b27] Handler A. M. & Harrell R. A. Germline transformation of *Drosophila melanogaster* with the *piggyBac* transposon vector. Insect Molecular Biology 8, 449–457 (1999). 10634970 10.1046/j.1365-2583.1999.00139.x

[b28] Coates C. J., Jasinskiene N., Miyashiro L. & James A. A. *Mariner* transposition and transformation of the yellow fever mosquito, *Aedes aegypti*. Proc Natl Acad Sci USA 95, 3748–3751 (1998). 9520438 10.1073/pnas.95.7.3748 PMC19908

[b29] Schetelig M. F. & Handler A. M. Germline transformation of the spotted wing drosophilid, Drosophila suzukii, with a piggyBac transposon vector. Genetica 141, 189–193, doi: 10.1007/s10709-013-9717-6 (2013). 23564446

[b30] Horn C., Schmid B. G., Pogoda F. S. & Wimmer E. A. Fluorescent transformation markers for insect transgenesis. Insect Biochemistry and Molecular Biology 32, 1221–1235 (2002). 12225913 10.1016/s0965-1748(02)00085-1

[b31] Schetelig M. F. & Handler A. M. A transgenic embryonic sexing system for *Anastrepha suspensa* (Diptera: Tephritidae). Insect Biochem Mol Biol 42, 790–795, doi: 10.1016/j.ibmb.2012.07.007 (2012). 22858603

[b32] Horn C. & Wimmer E. A. A versatile vector set for animal transgenesis. Development Genes and Evolution 210, 630–637 (2000). 11151300 10.1007/s004270000110

[b33] Ashburner M. Drosophila A Laboratory Manual. Vol. 2 (Cold Spring Harbor Laboratories, 1989).

[b34] Potter C. J. & Luo L. Splinkerette PCR for mapping transposable elements in *Drosophila*. PLoS One 5, e10168, doi: 10.1371/journal.pone.0010168 (2010). 20405015 PMC2854151

[b35] Bellen H. J. . The BDGP gene disruption project: single transposon insertions associated with 40% of Drosophila genes. Genetics 167, 761–781, doi: 10.1534/genetics.104.026427 (2004). 15238527 PMC1470905

[b36] R Development Core Team. R: A language and environment for statistcal computing. R Foundation for Statistical Computing, Vienna, Austria. http://www.R-project.org/” (2016).

[b37] Bates D. M., Bolker M., Walker B. & Fitting S. Linear Mixed-Effects Models Using lme4. Journal of Statistical Software 67, 1–48, doi: 10.18637 (2015).

[b38] Fox J. & Weisberg S. An R Companion to Applied Regression. second edn (Sage Publications, Thousand Oaks CA, 2011).

[b39] Halekoh U. & Højsgaard S. A Kenward-Roger Approximation and Parametric Bootstrap Methods for Tests in Linear Mixed Models - The R Package pbkrtest. Journal of Statistical Software 59, 1–30 (2014).26917999

[b40] Scheipl F., Greven S. & Kuechenhoff H. Size and power of tests for a zero random effect variance or polynomial regression in additive and linear mixed models. Computational Statistics & Data Analysis 52, 3283–3299 (2008).

[b41] A Package for Survival Analysis in S. Version 2.38 (2015).

[b42] Hothorn T., Bretz F. & Westfall P. Simultaneous Inference in General Parametric Models. Biometrical Journal 50, 346–363 (2008). 18481363 10.1002/bimj.200810425

[b43] Horn M. & Vollandt R. Multiple Tests und Auswahlverfahren(Gustav Fischer Verlag, 1995).

[b44] Fleiss J. L., Levin B. & M. C. Paik Statistical Methods for Rates and Proportions(Wiley, 2003).

