# Supplementary material for: Cre/lox-Recombinase-Mediated Cassette Exchange for Reversible Site-Specific Genomic Targeting of the Disease Vector, Aedes aegypti
Source: Sci Rep. 2017 Mar 7;7:43883. doi: 10.1038/srep43883 (PMC5339718; doi:10.1038/srep43883)
Supplement: Supplementary Information [file srep43883-s1.pdf]

Supplementary Information

Cre/*lox*-Recombinase-Mediated Cassette Exchange for Reversible Site-Specific Genomic Targeting of the Disease Vector, *Aedes aegypti*

Authors:

Irina Häcker[1*], Robert Harrell II[2,3], Gerrit Eichner[4], Kristina L. Pilitt[2], David O'Brochta[2,5], Alfred M. Handler[6], Marc F. Schetelig[1*]

Affiliations:

1 Institute for Insect Biotechnology, Justus-Liebig-University Giessen, Heinrich-Buff-Ring 26-32, 35392 Giessen, Germany

2 Institute for Bioscience and Biotechnology Research, University of Maryland, College Park, Rockville, Maryland 20850

3 Insect Transformation Facility, University of Maryland, College Park, Rockville, Maryland 20850

4 Mathematical Institute, Justus-Liebig-University Giessen, Arndtstrasse 2, 35392 Giessen, Germany

5 Department of Entomology, University of Maryland, College Park, Rockville, Maryland 20850

6 USDA/ARS, Center for Medical, Agricultural and Veterinary Entomology, 1700 SW 23rd Drive, Gainesville, FL 32608, USA

* Corresponding authors

Supplementary Figure S1

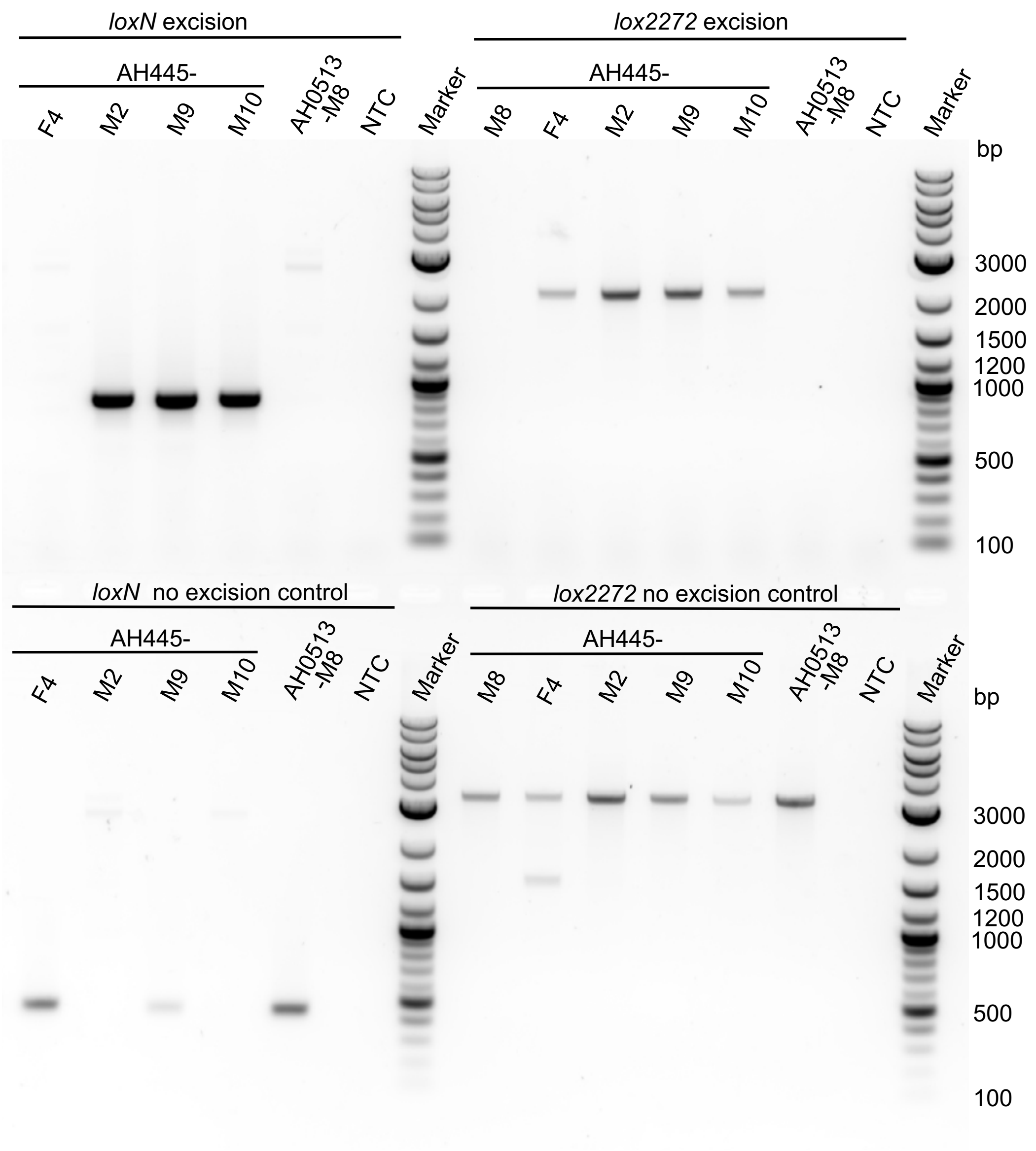

Supplementary Figure S1. Agarose gel showing PCR reactions to prove *loxN* and *lox2272* excisions as well as no excision control reactions. *loxN* excision was phenotypically visible by loss of cyan marker expression. Several pupae with only red eyes were pooled exemplary for 3 families, AH445-M2, -M9, -M10. PCR was conducted with primers spanning the *loxN* integration site, creating an 840 bp product in case of correct *loxN* excision, and more than 5.6 kb in case of no excision (AH0513-M8 control). As an independent control ("*loxN* no excision control") served a PCR reaction using a primer on the donor plasmid backbone, such that a PCR product can only be obtained if *loxN* excision did not occur (AH445-F4, AH0513-M8). The faint positive band for AH445-M9 originates from a contamination of the pool of red-eyed pupae with an individual without *loxN* excision (red and cyan eyes). The *lox2272* excision panel shows the positive PCR reactions proving *lox2272* excision for 4 of the 6 positively tested families (tested were pools of red and cyan-eyed pupae of each of the 16 G_0 families producing offspring; Table S2). The *lox2272* no excision control was positive for all tested families, as the pools contained pupae positive and negative for lox2272 excision. NTC denotes the no template control reaction for each primer pair. Marker denotes the 2-log ladder from NEB. bp = base pairs

Supplementary Figure S2.

A) Female fecundity

1)

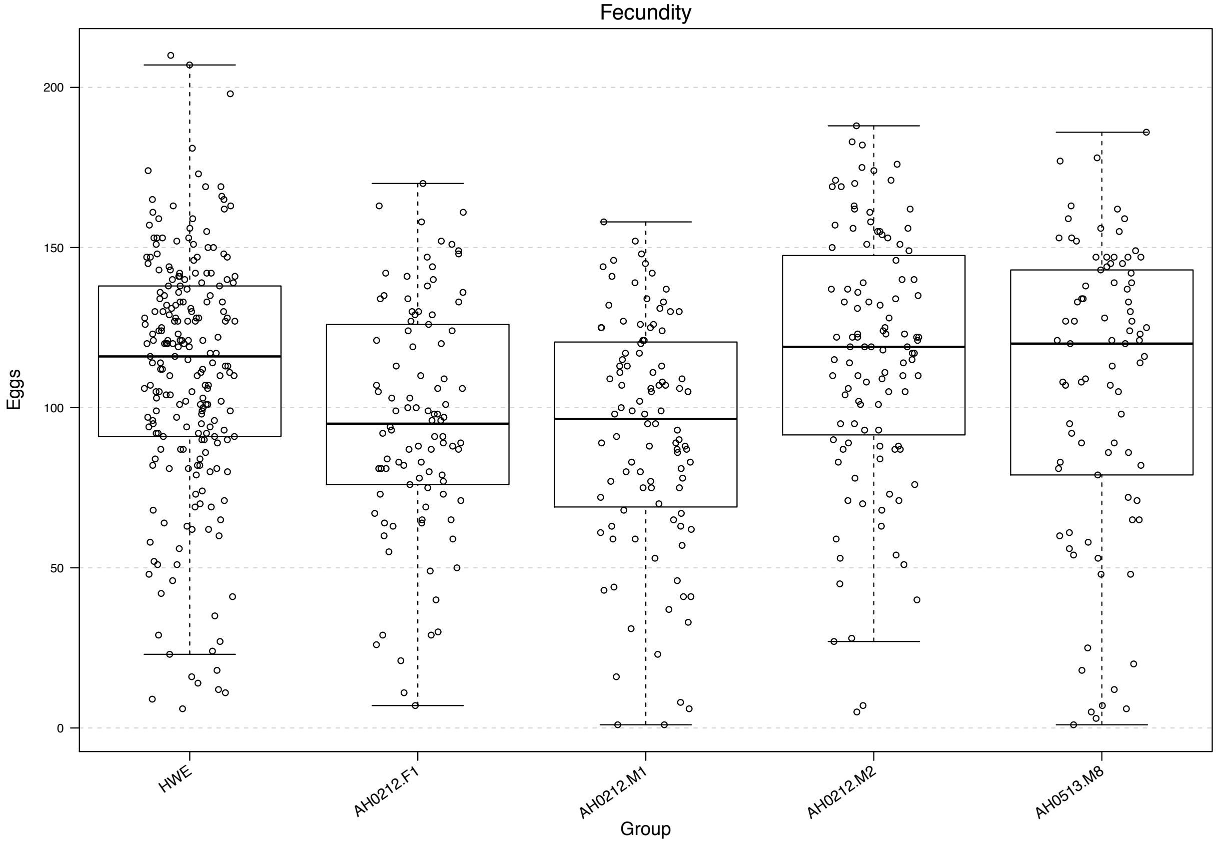

2)

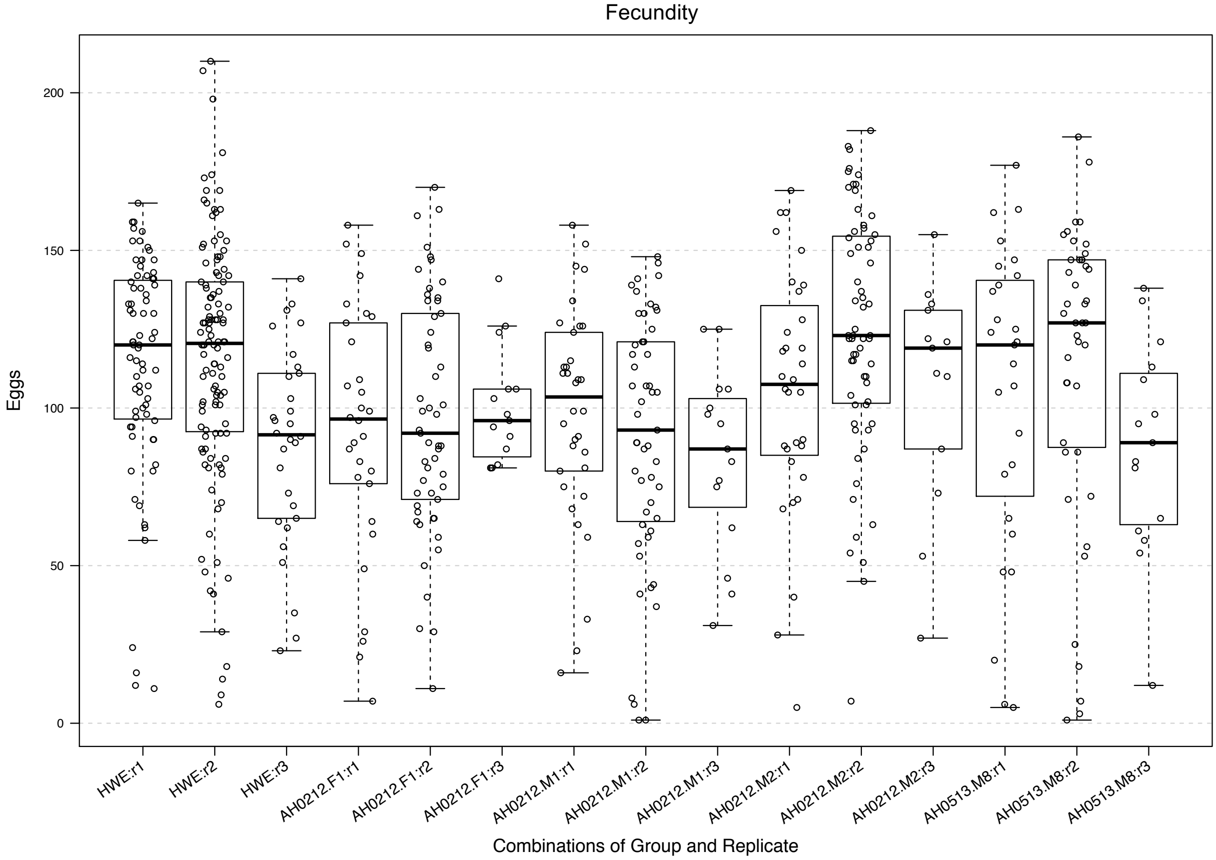

B) Female fertility with fish food

1)

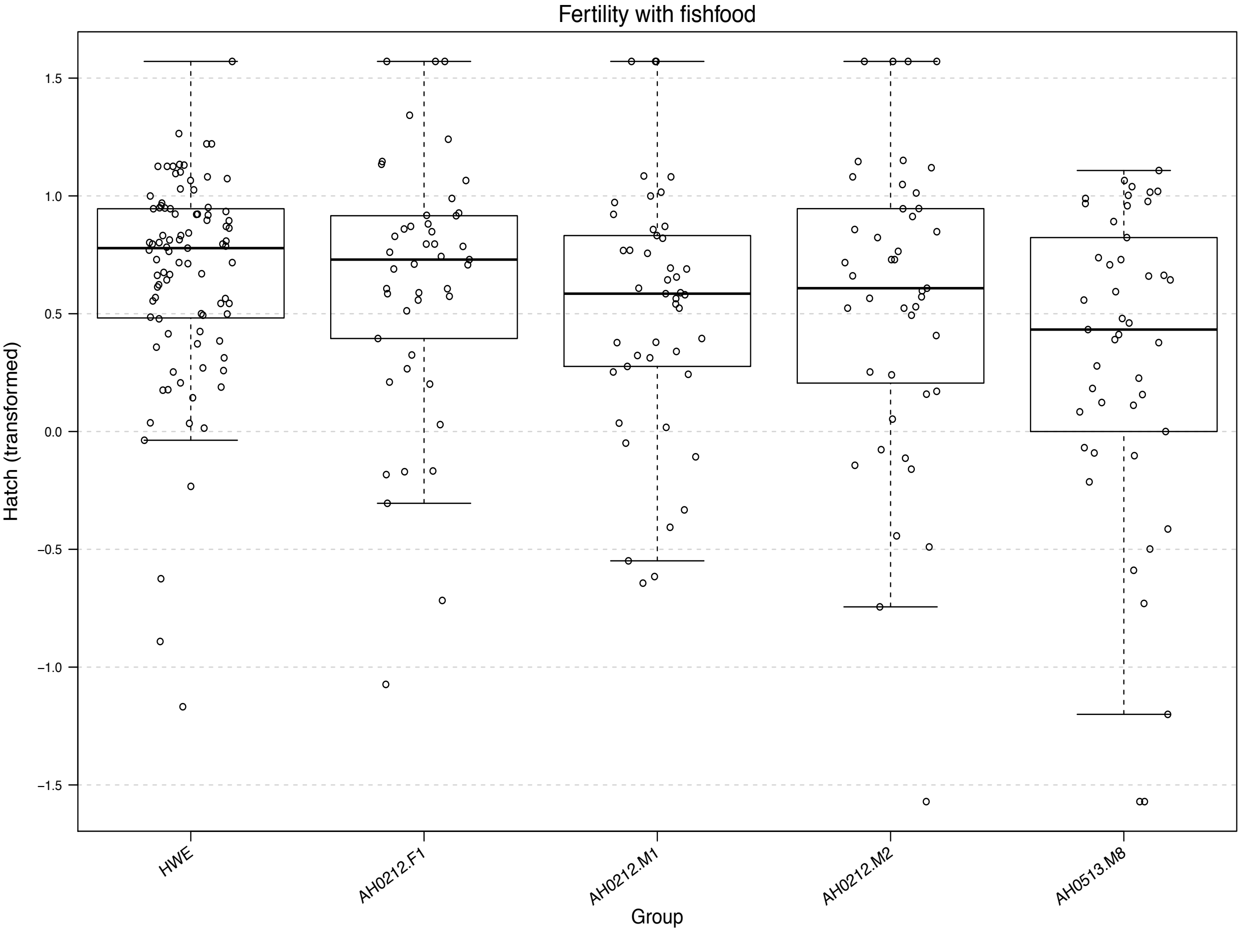

2)

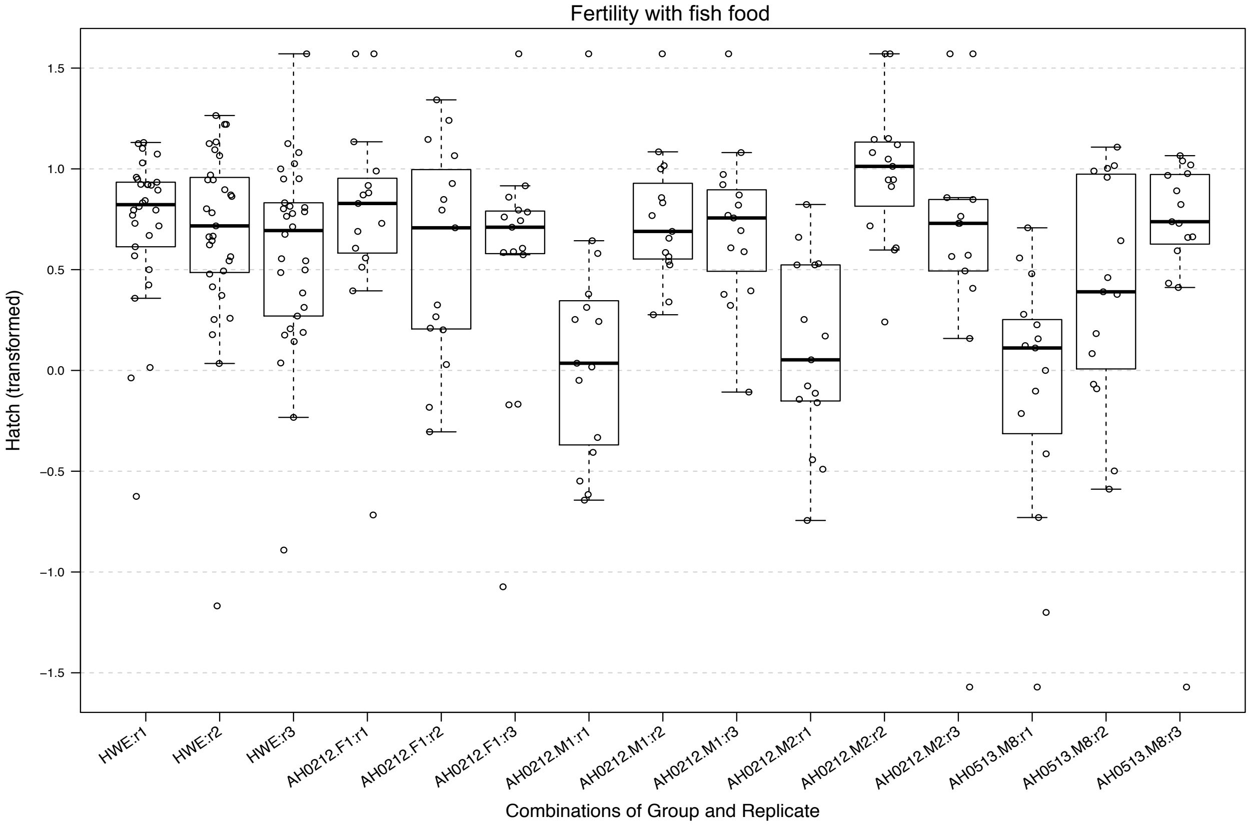

C) Female fertility with yeast

1)

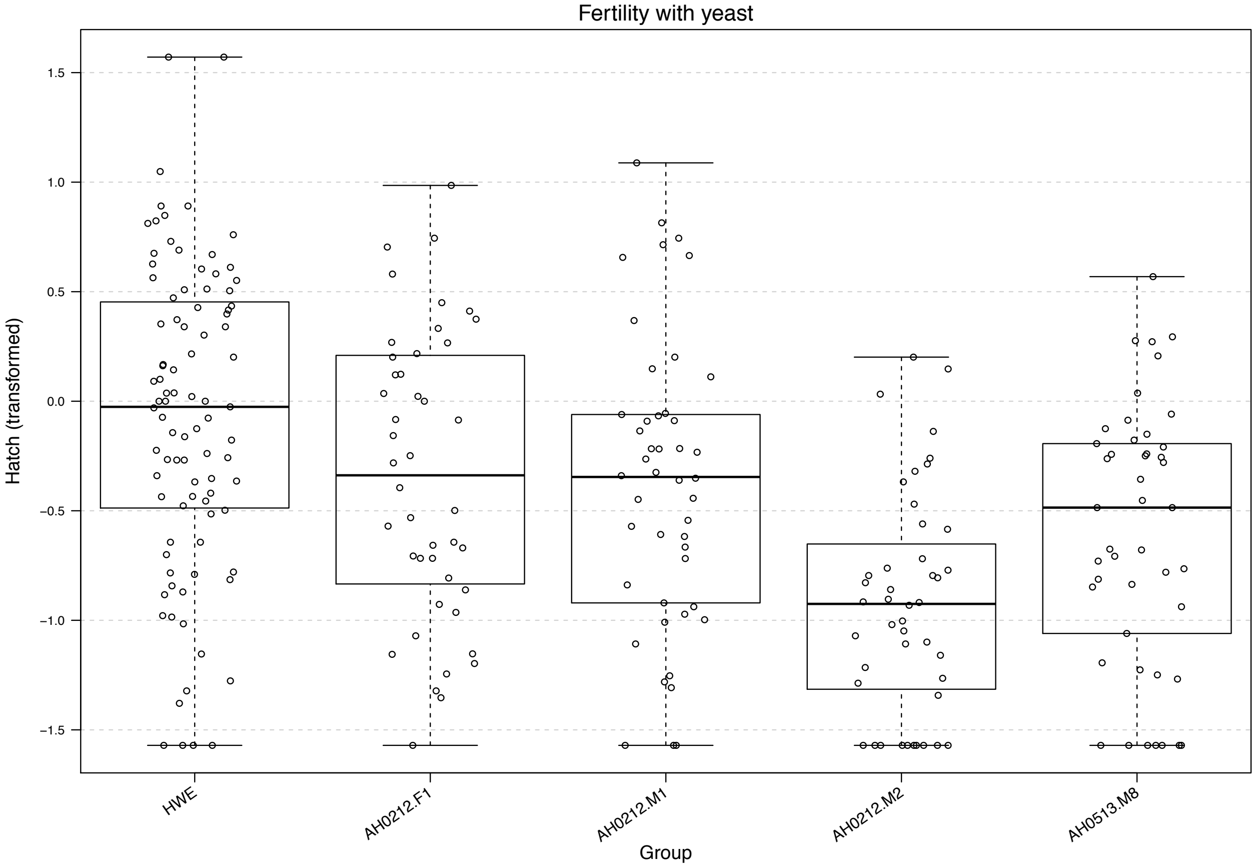

2)

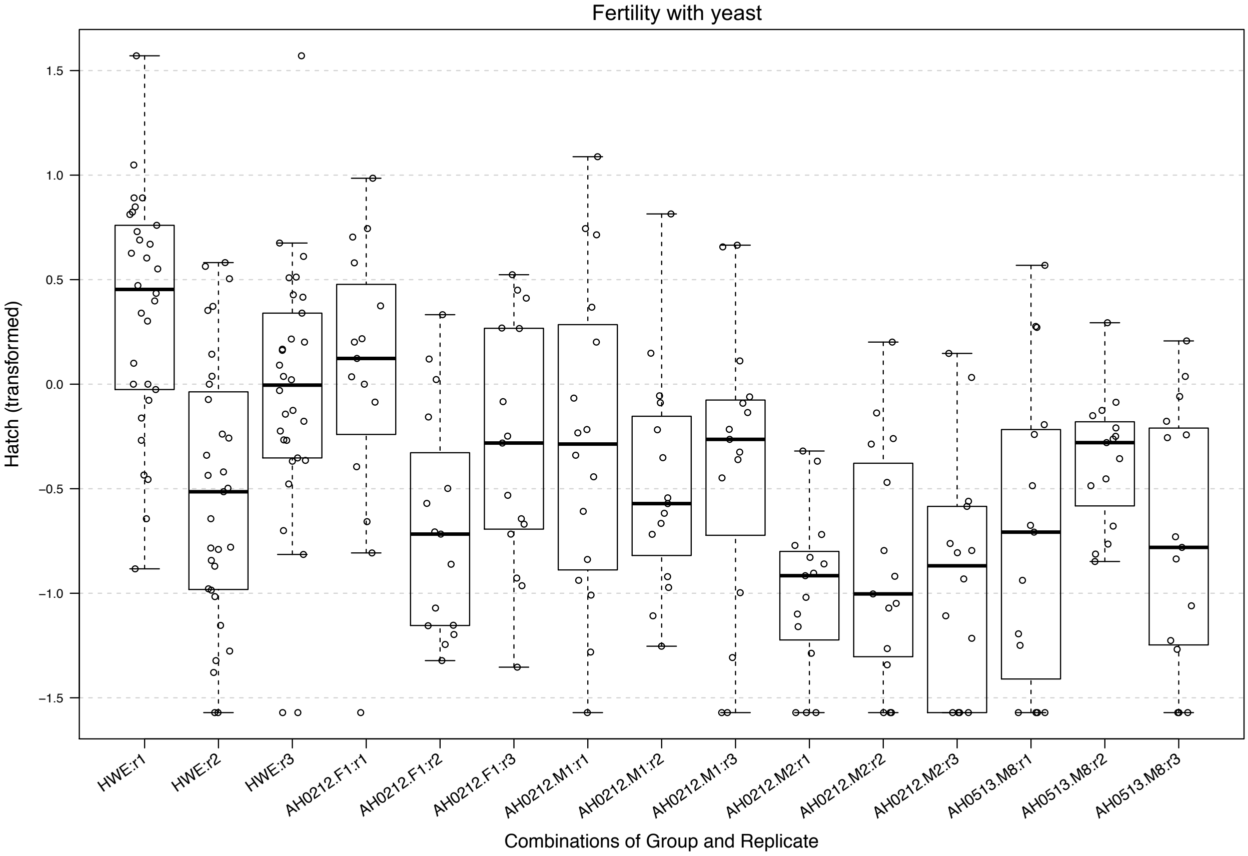

D) Larval development time

1)

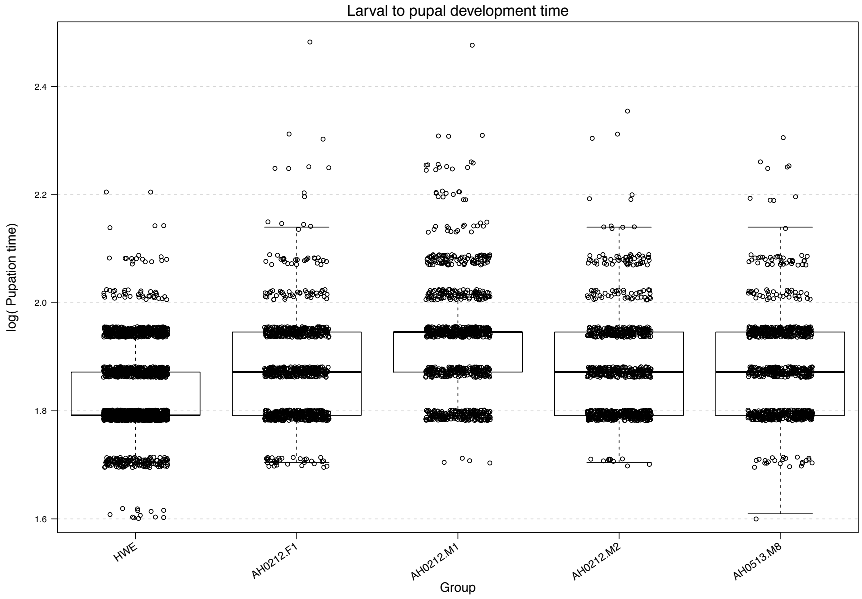

2)

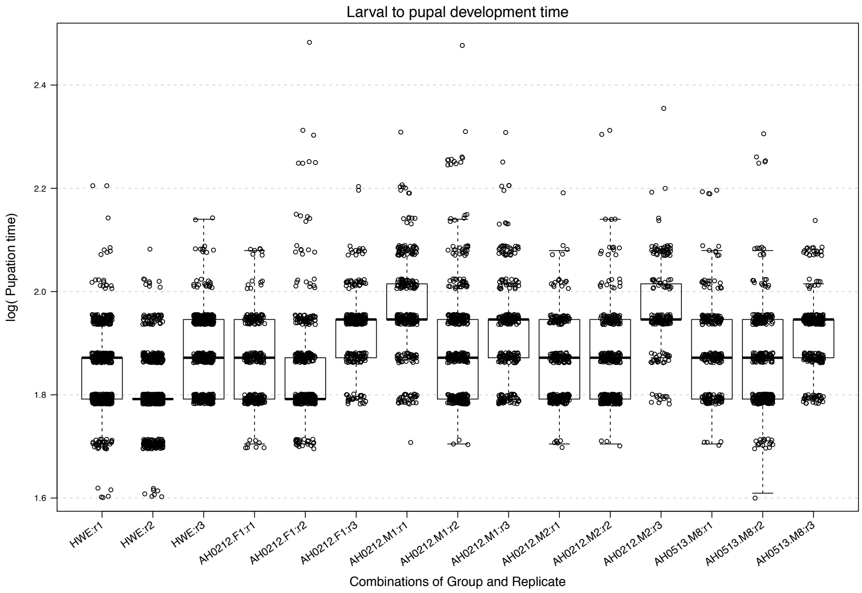

3)

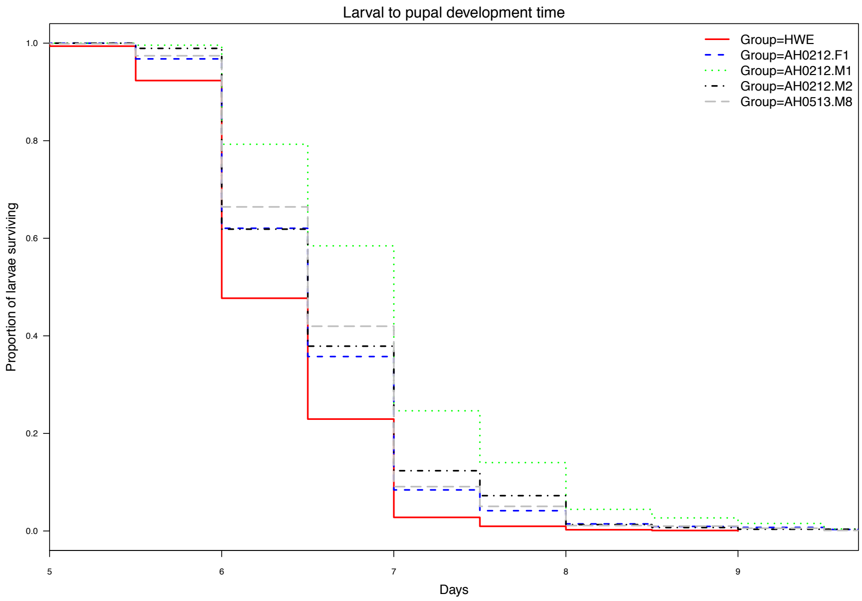

E) Female adult longevity

1)

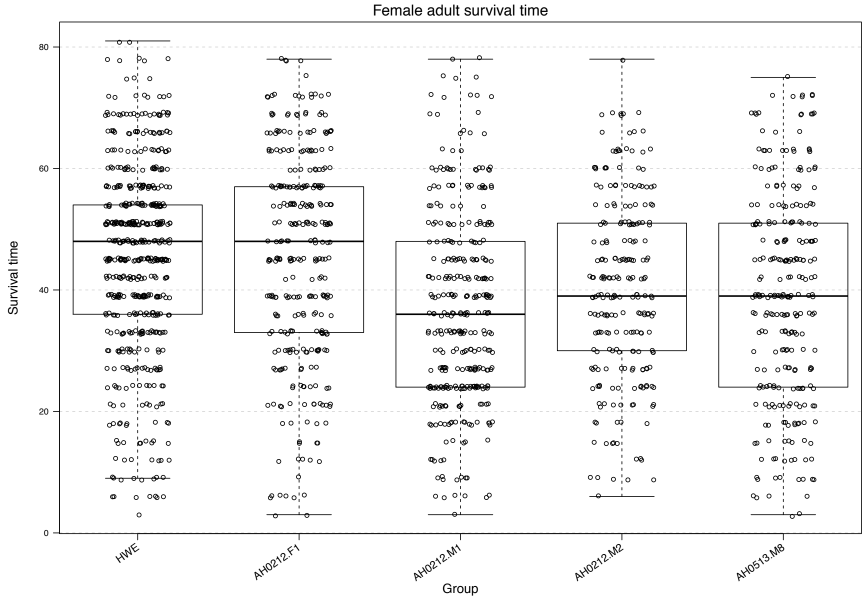

2)

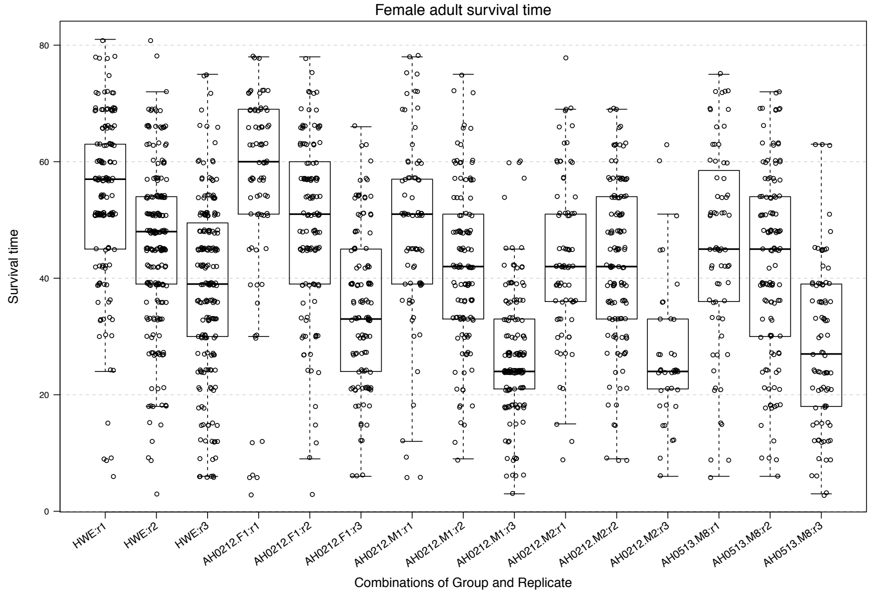

3)

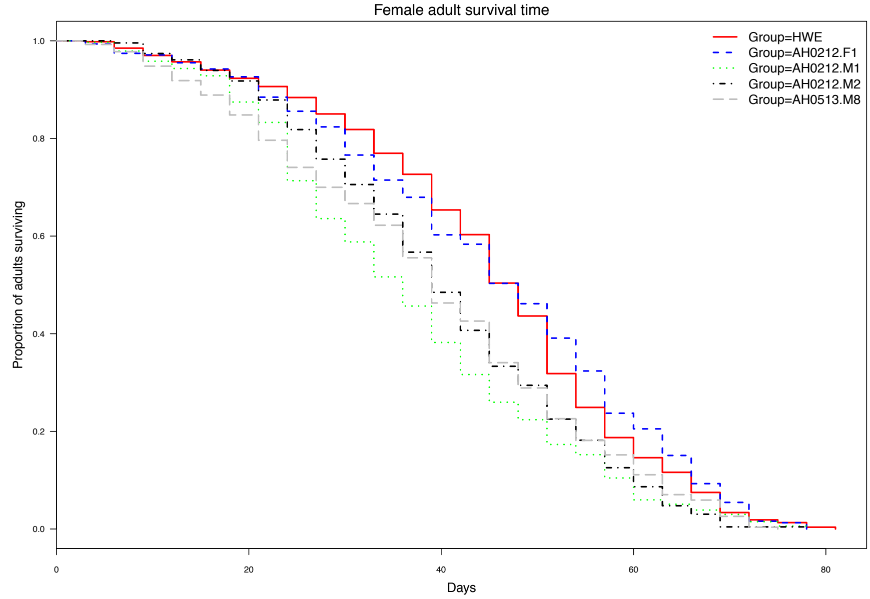

F) Male adult longevity

1)

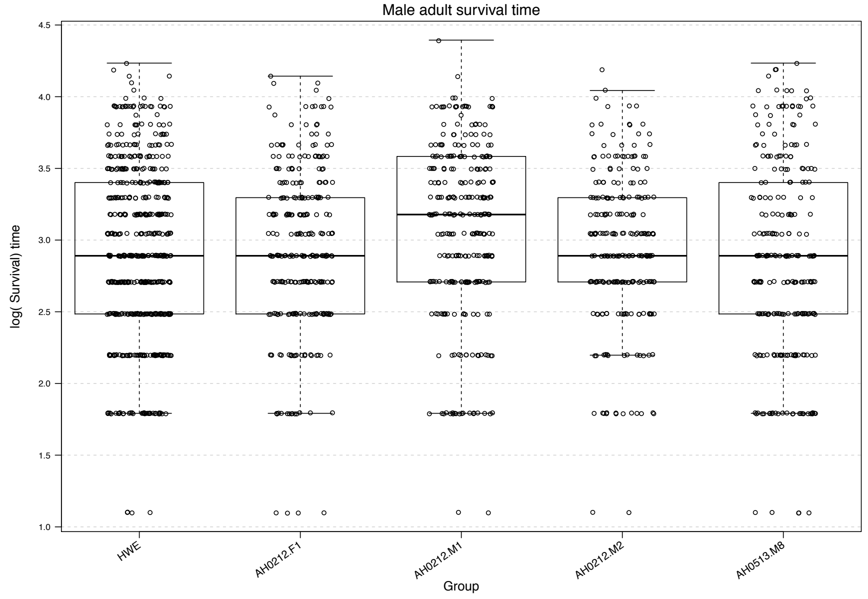

2)

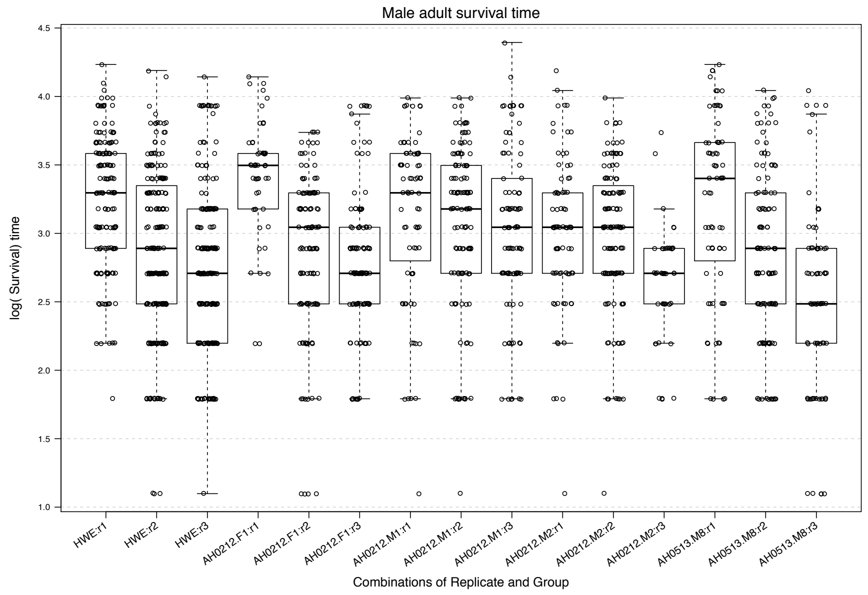

3)

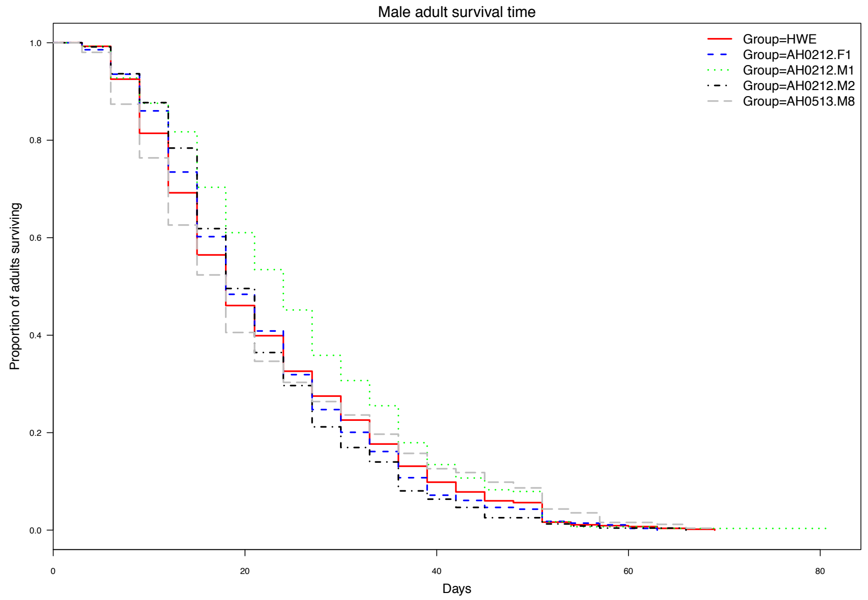

Supplementary Figure S2. Combination of box plots und scatter plots to visualize populations means and the variation in samples for **A)** Female fecundity, **B)** Female fertility, **C)** Larval development time, **D)** Female longevity, **E)** Male longevity.

Means and sample variation are shown as the average of all replicates **(1)** or by replicate and line **(2)**. For larval development and adult longevity tests also the survival curves are shown **(3)**.

Supplementary Figure S3.

A) Female fecundity

1)

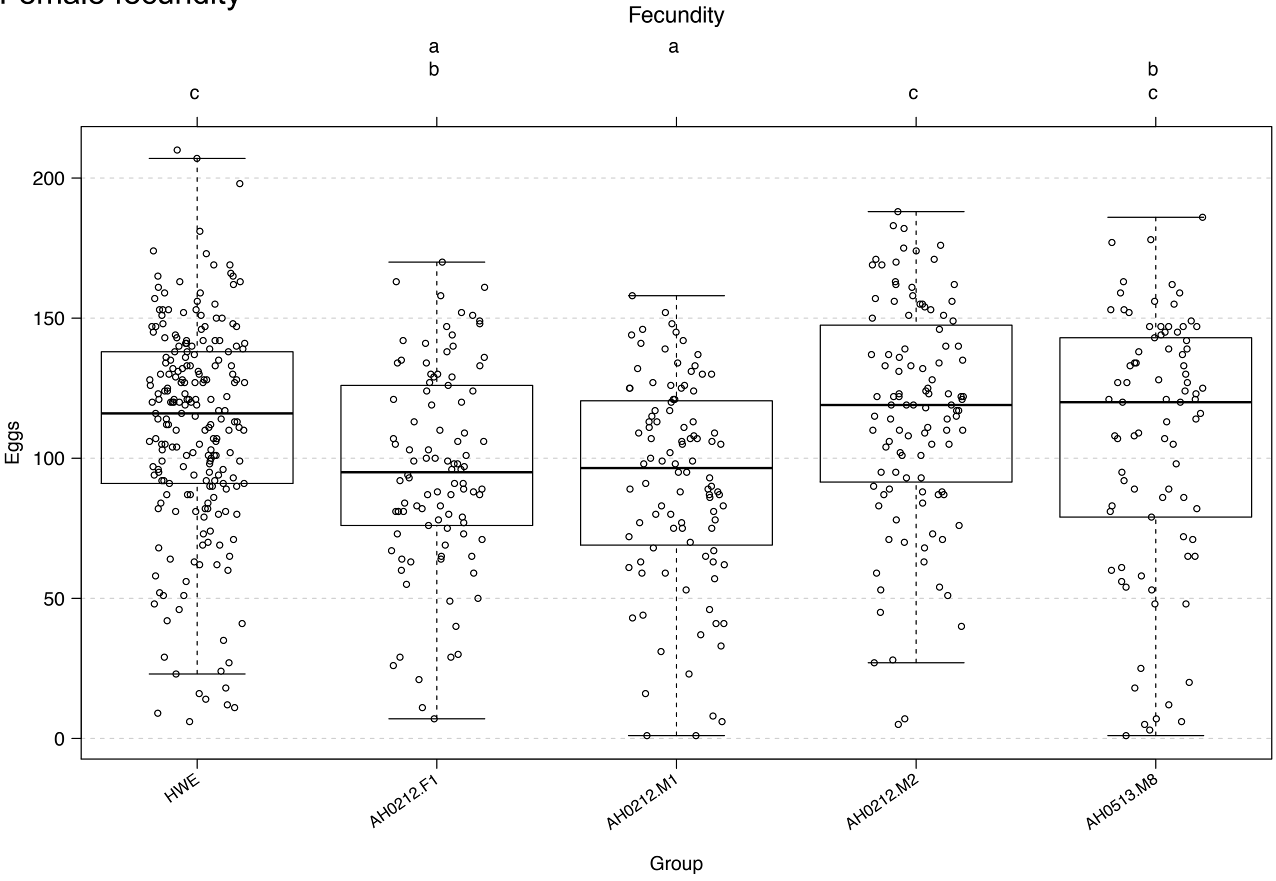

2)

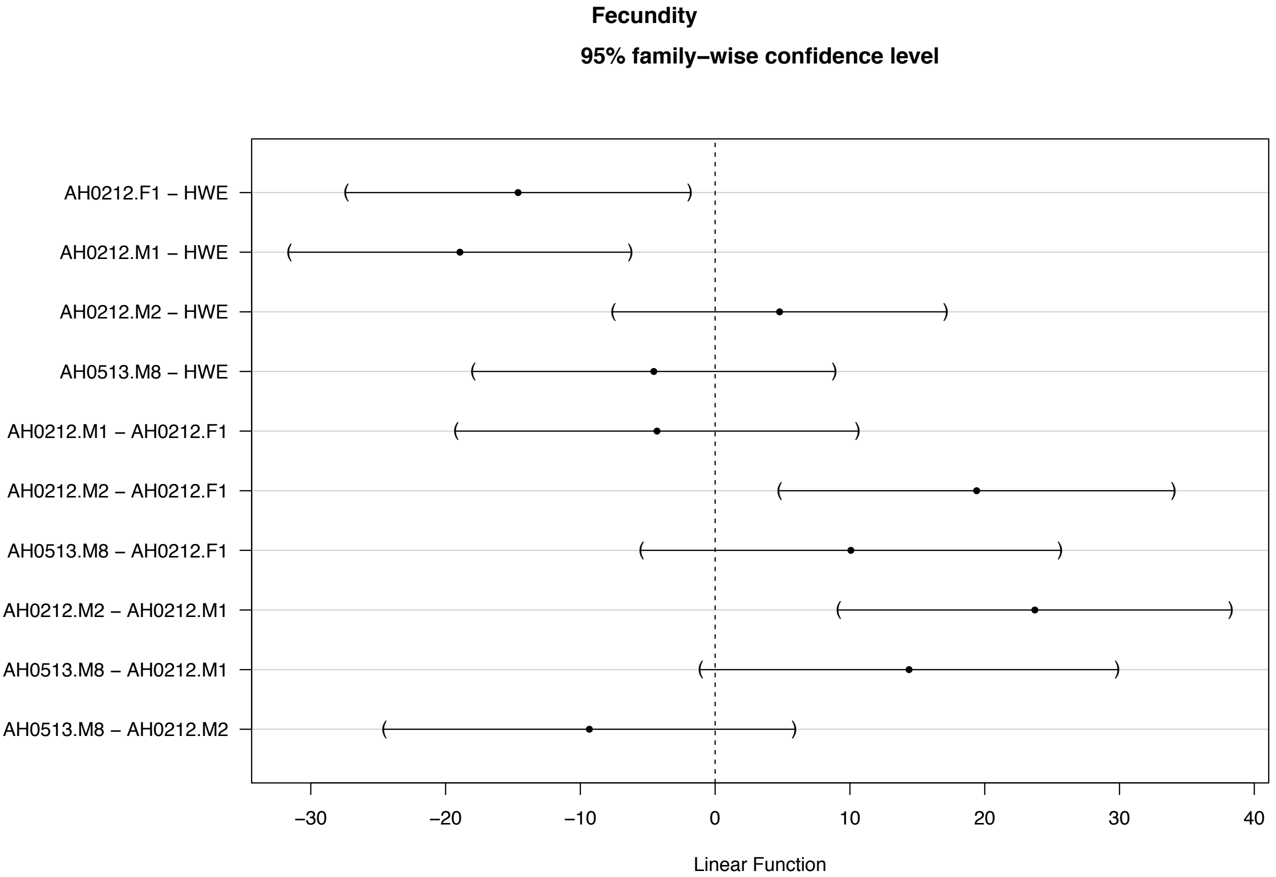

B) Female fertility with fish food

1)

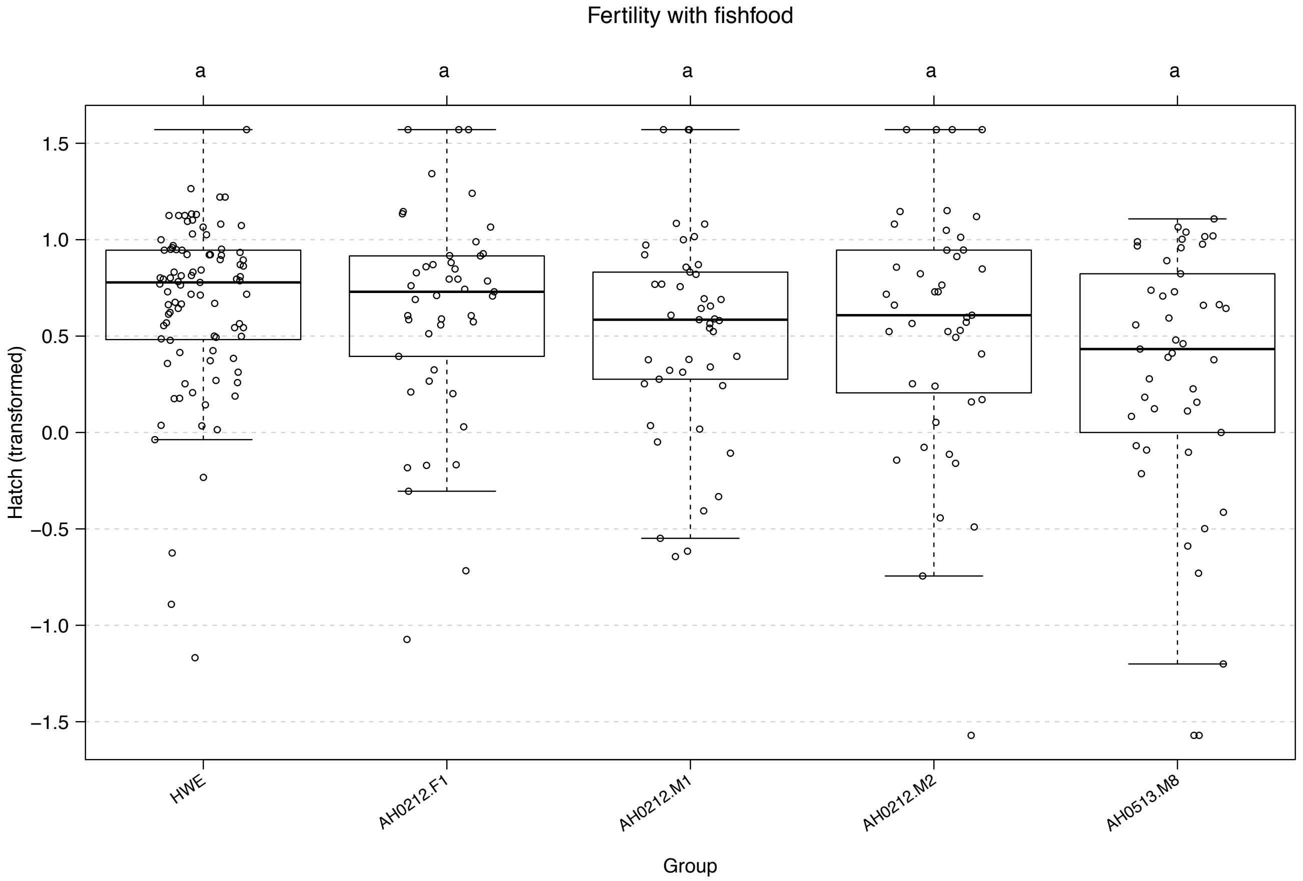

2)

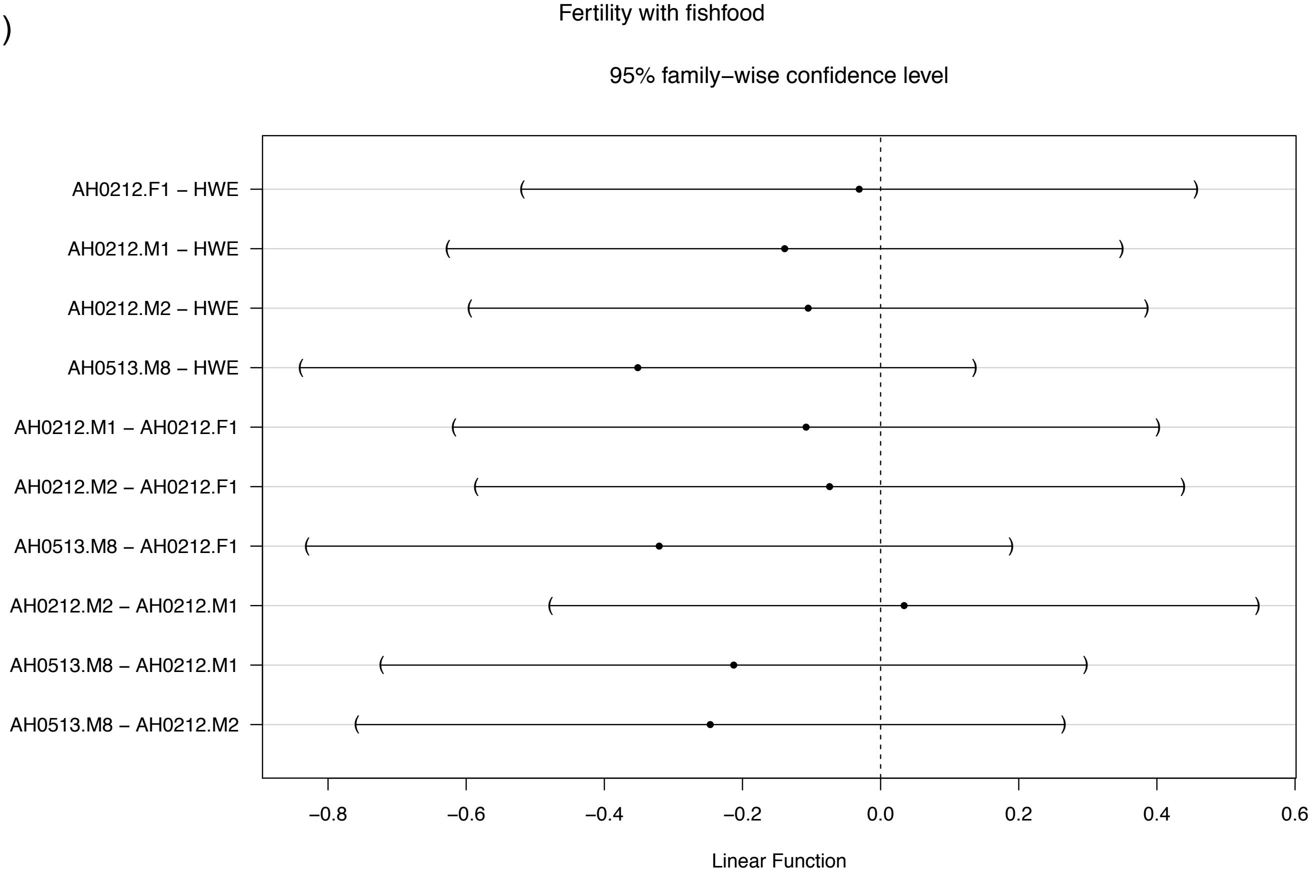

B) Female fertility with yeast

1)

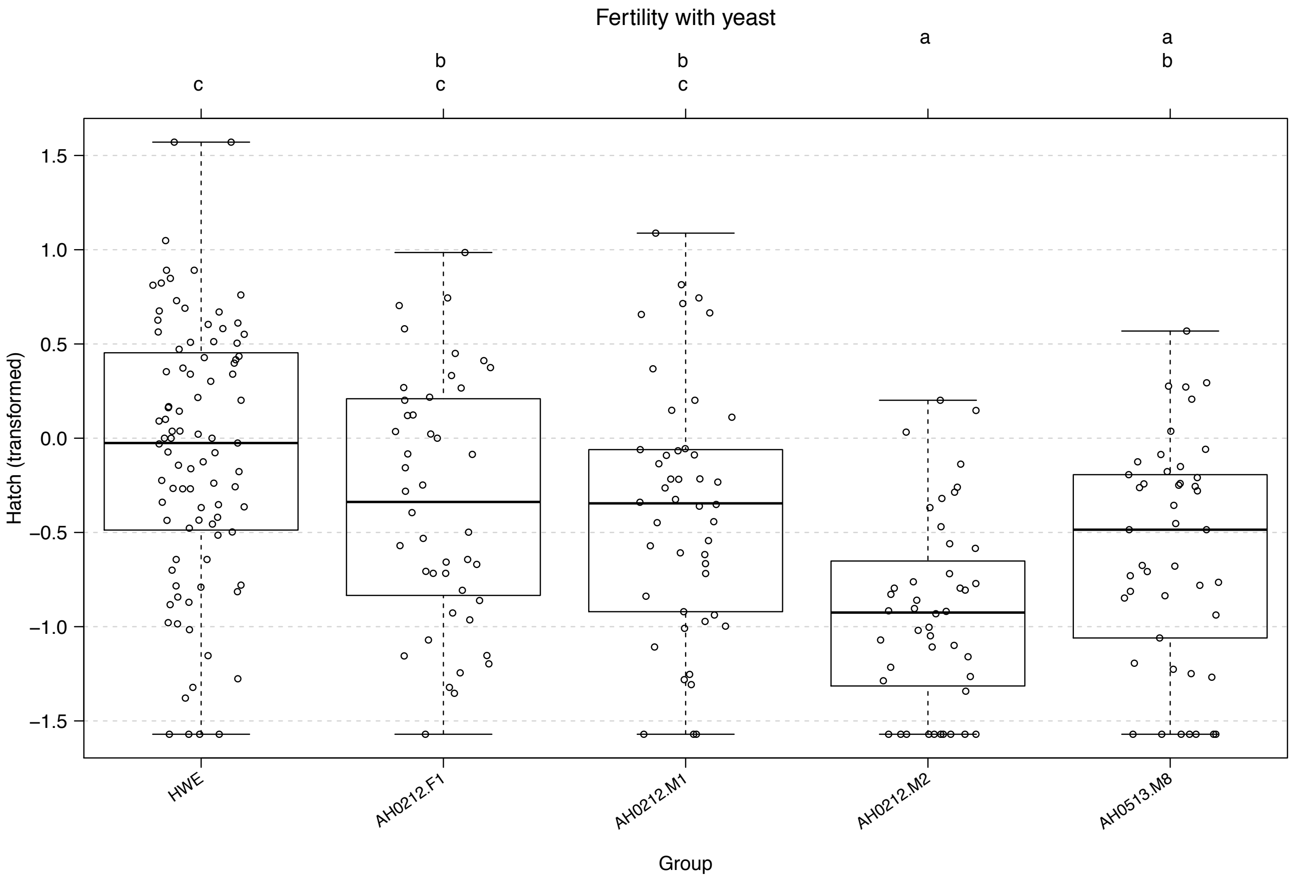

2)

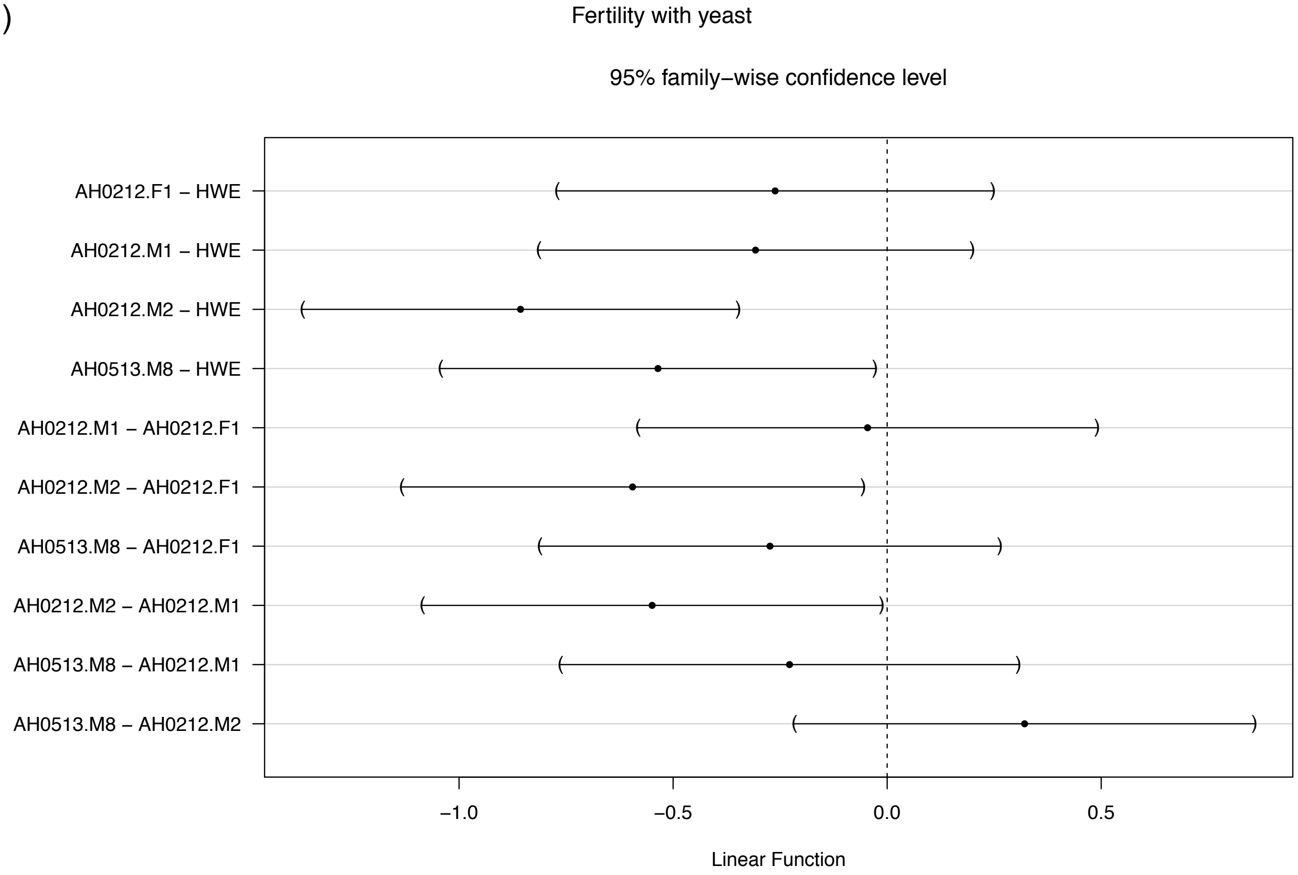

C) Larval development time

1)

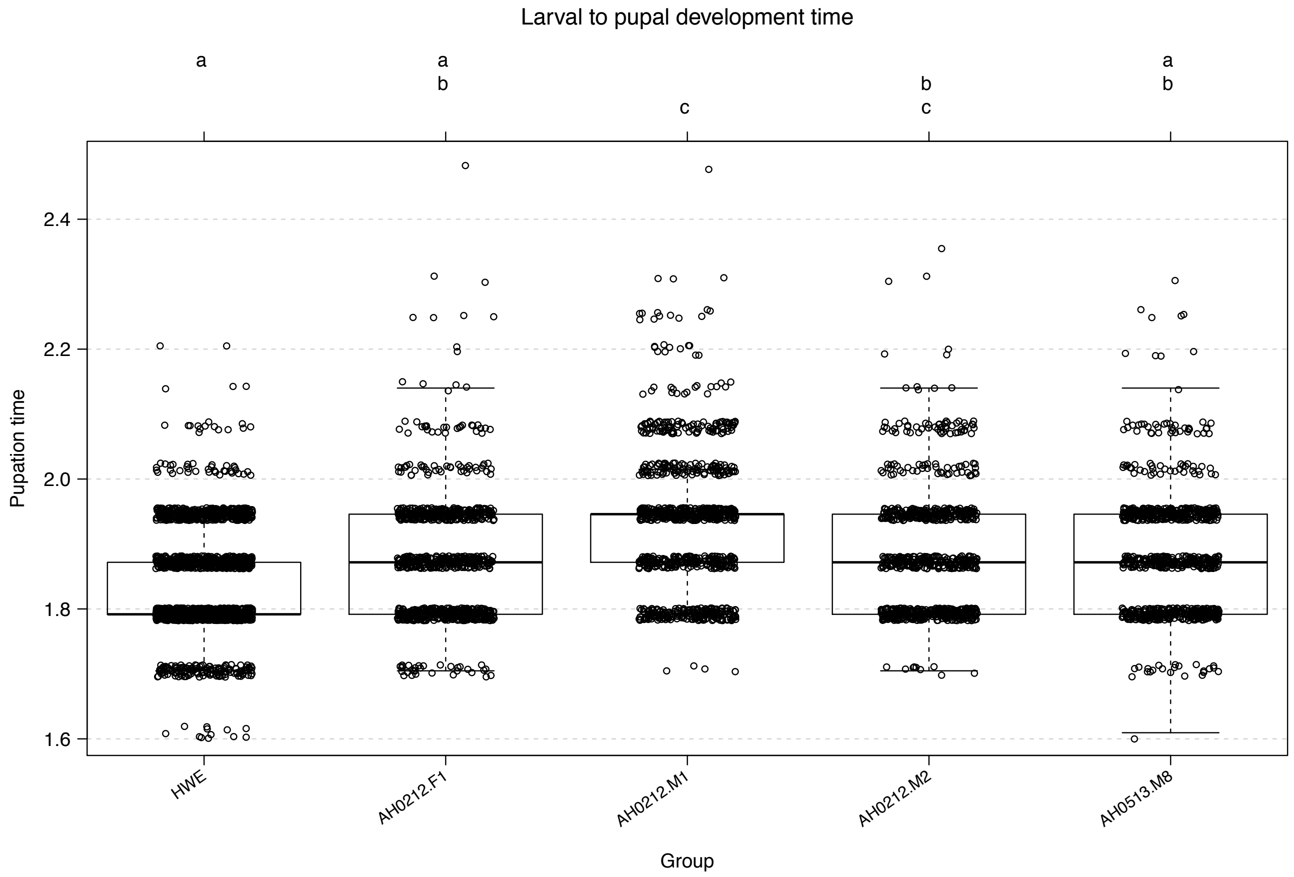

2)

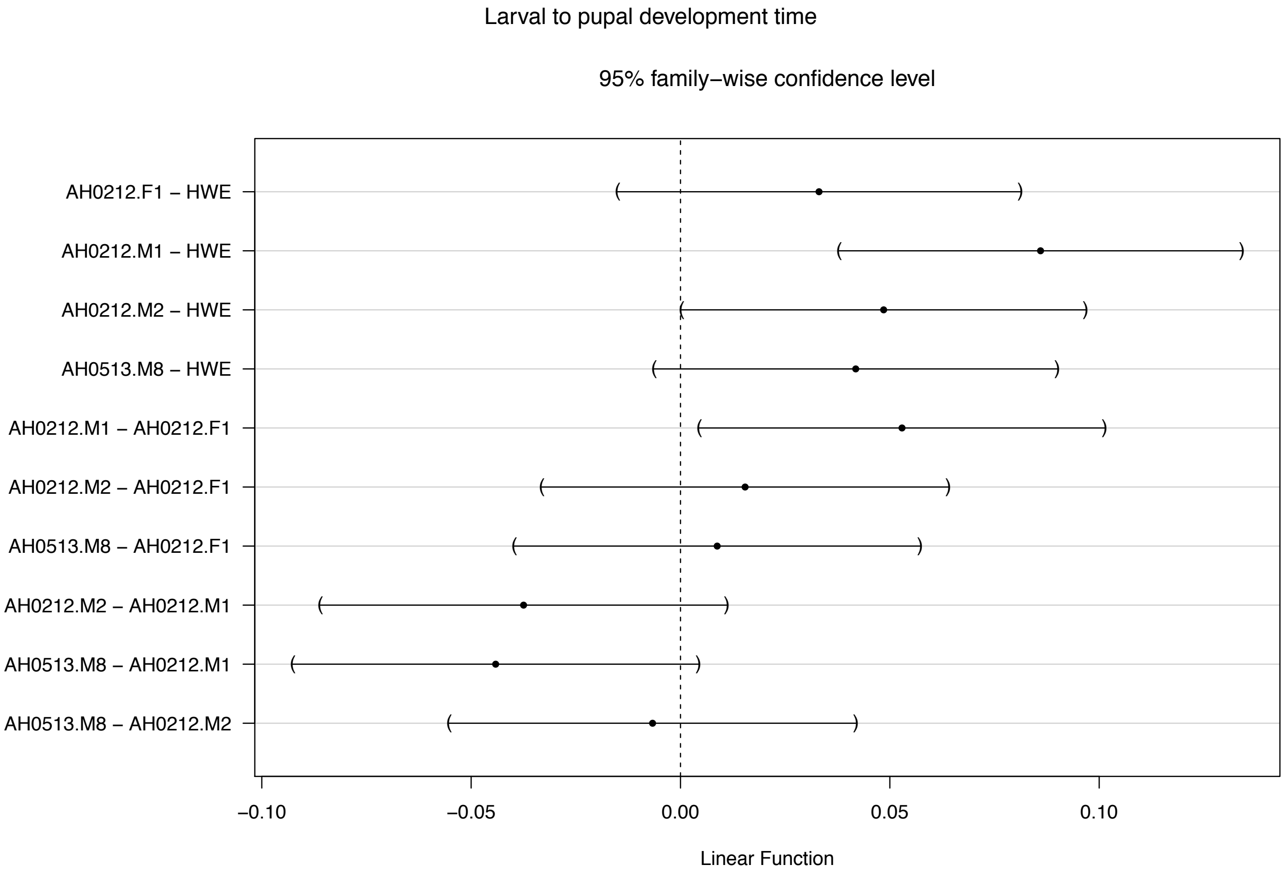

D) Female longevity

1)

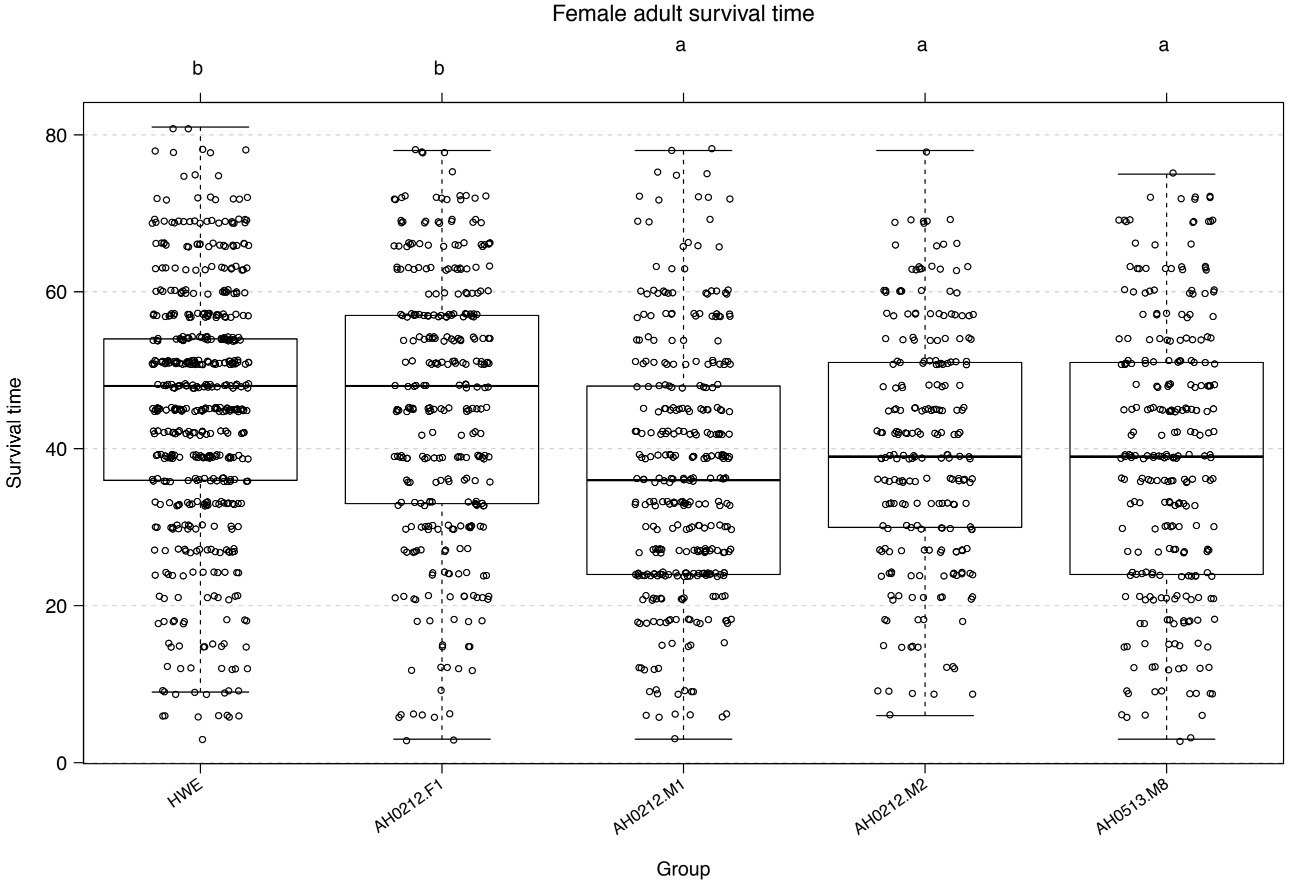

2)

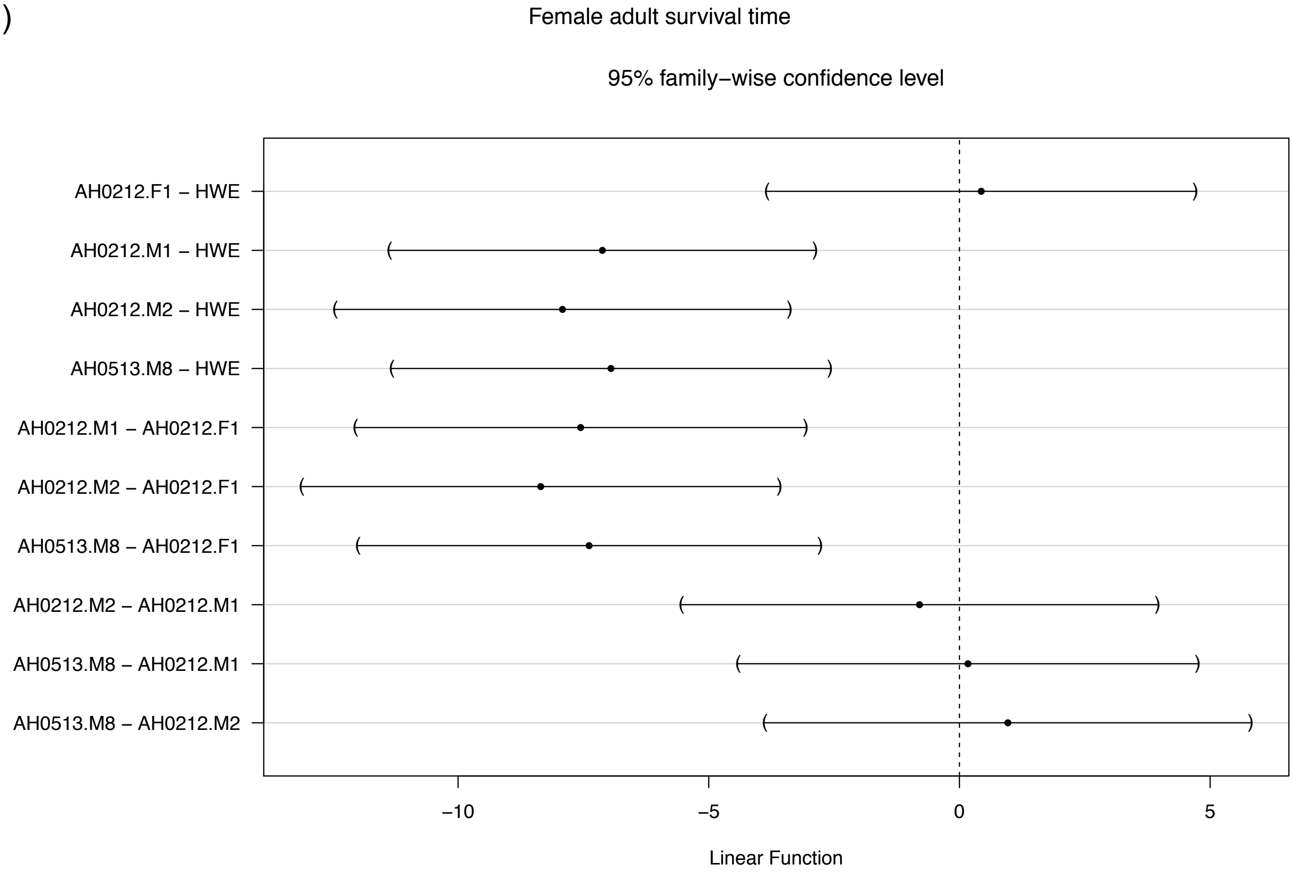

D) Male longevity

1)

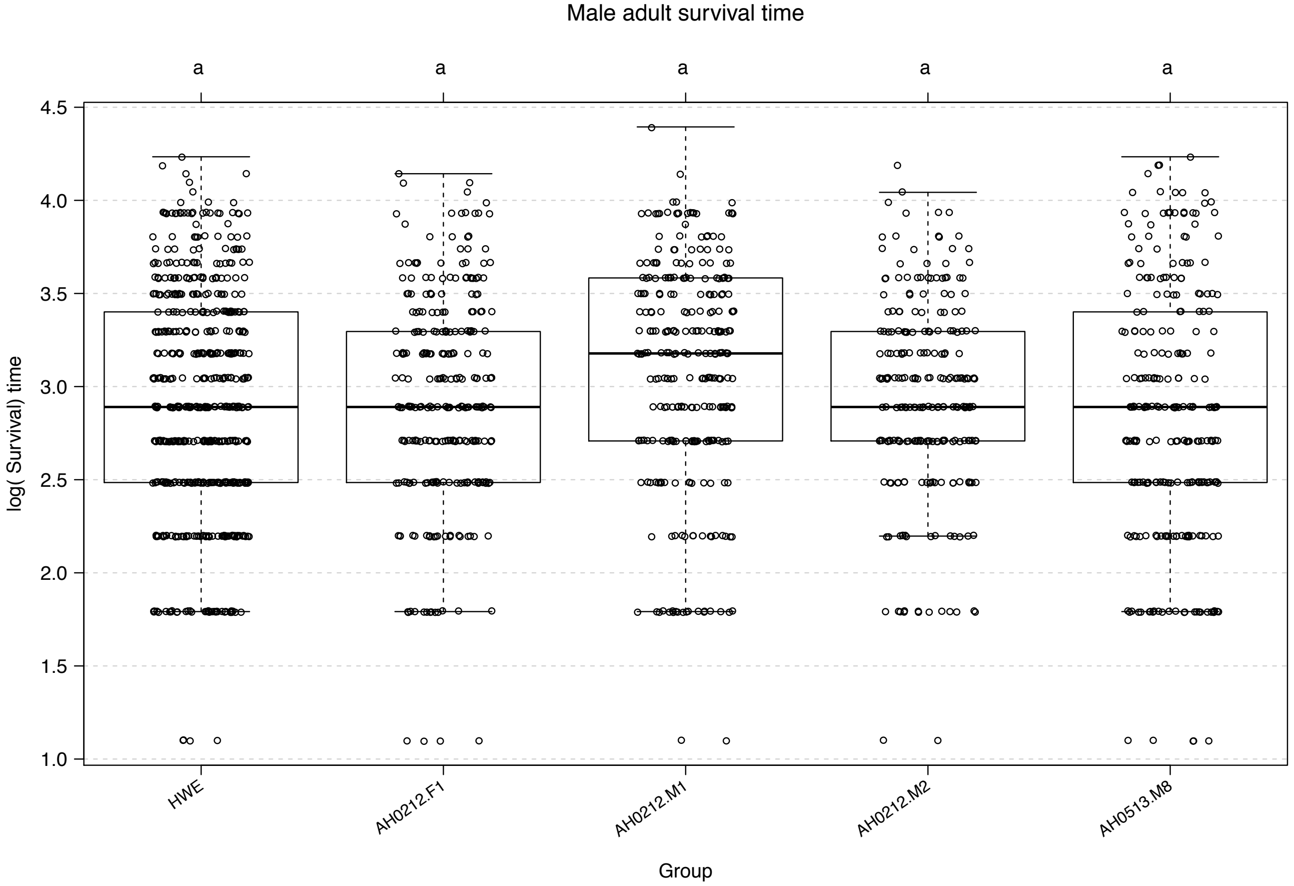

2)

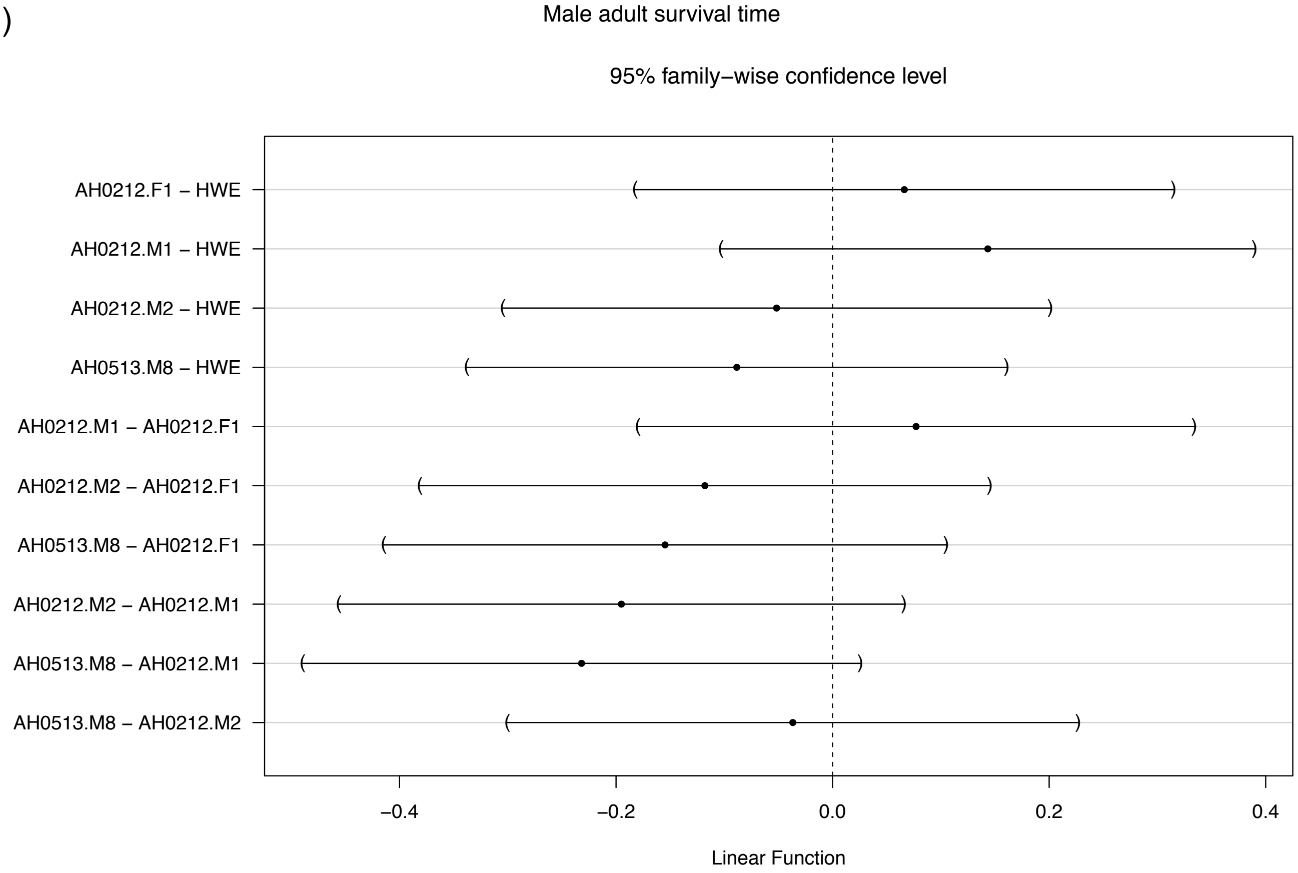

Supplementary Figure S3. Analysis of differences between lines using linear mixed effect models. The line (i.e. genomic integration site) is considered as fixed effect, the replicate and the interaction between replicate and line as random effects. **A)** Female fecundity, **B)** Female fertility, **C)** Larval development time, **D)** Female longevity, **E)** Male longevity. For each test, significant differences of means (Tukey's 2-sided multiple comparisons of all means with p-values adjusted by Westfall's truncated closed stepwise procedure) are indicated by compact letter display above box plots **(1)**. Moreover, a plot of the two-sided simultaneous confidence intervals for the Tukey test (of all pairwise comparisons of means, adjusted by a single-step method as simultaneous confidence intervals do not exist for Westfall's stepwise procedure) with a family-wise confidence level of 95% is shown **(2)**. Each pair of groups whose (solid-bold horizontal) interval does not contain zero (i.e., which does not intersect the dashed vertical line) has significantly different group means. All results shown consider the possible interaction between line and replicate.

Supplementary Table S2.

Results of 2nd step RMCE experiments. *loxN-loxN* recombination results in a red-eyed phenotype. *Lox2272-lox2272* recombination results in unchanged red- and cyan-eyed phenotype that cannot be distinguished from the parental line AH0513-M8. Exemplary *loxN-loxN* recombinations were confirmed by PCR. *Lox2272-lox2272* recombination was analyzed by PCR for each family.

No.	**Name G_0 family**	**Total G_1 number screened for phenotypical change**	**Number G_1 with red eye phenotype (*loxN* excision)**	**Number G_1 with red and cyan eye phenotype (*lox2272* excision or no event)**	**% *loxN* excision**	**proof *loxN* excision by PCR**	**proof *lox2272* excision by PCR**
1	**AH445-F1**	3	0	3	0.0	not analyzed	negative
2	**AH445-F2**	303	2	301	0.7	not analyzed	negative
3	**AH445-F3**	647	2	645	0.3	not analyzed	negative
4	**AH445-F4**	27	0	27	0.0	not analyzed	confirmed
5	**AH445-F5**	no offspring					
6	**AH445-F6**	no offspring					
7	**AH445-F7**	48	0	48	0.0	not analyzed	negative
8	**AH445-M1**	300	18	282	6.0	not analyzed	confirmed
9	**AH445-M2**	406	130	276	32.0	confirmed	confirmed
10	**AH445-M3**	514	0	514	0.0	not analyzed	negative
11	**AH445-M4**	273	0	273	0.0	not analyzed	negative
12	**AH445-M5**	594	15	579	2.5	not analyzed	confirmed
13	**AH445-M6**	386	0	386	0.0	not analyzed	negative
14	**AH445-M7**	509	0	509	0.0	not analyzed	negative
15	**AH445-M8**	158	1	157	0.6	not analyzed	negative
16	**AH445-M9**	486	61	425	12.6	confirmed	confirmed
17	**AH445-M10**	395	15	380	3.8	confirmed	confirmed
18	**AH445-M11**	186	0	186	0.0	not analyzed	negative

Supplementary Table S3.

Results of the statistical analysis of fitness test data (fecundity, fertility, larval development, and adult longevity). Shown are the results of Tukey's 2-sided multiple comparisons (of means) for all. For each test, the analysis was once performed without considering the possible effect of the interaction between replicate and line, and once considering the effect. Although the tests were not designed to detect such interactions, the latter values were used in the test evaluation to not potentially overestimate the difference between lines. P values were adjusted for multiple comparisons either with the single step method and corresponding, equivalent simultaneous confidence intervals for all differences in means were computed (results visualized in Supplementary Figure S2). In addition, Westfall's truncated closed stepwise procedure for Tukey's two-sided multiple comparisons of all means – a more powerful extension of the single-step method – was applied to potentially increase the number of significant differences. Its results were visualized by a "compact letter display" (Supplementary Figure S2).
Significant codes: 0 ‘***’ 0.001 ‘**’ 0.01 ‘*’ 0.05 ‘.’ 0.1 ‘ ’ 1

1) Fecundity

- Interaction between replicate and strain: not considered
- P value adjustment: single-step method

```
Linear Hypotheses:
                           Estimate Std. Error z value Pr(>|z|)
AH0212.F1 - HWE == 0        -14.620      4.684  -3.121  0.01527 *
AH0212.M1 - HWE == 0        -18.936      4.651  -4.071  < 0.001 ***
AH0212.M2 - HWE == 0          4.783      4.533   1.055  0.82707
AH0513.M8 - HWE == 0         -4.549      4.926  -0.923  0.88624
AH0212.M1 - AH0212.F1 == 0   -4.316      5.474  -0.788  0.93297
AH0212.M2 - AH0212.F1 == 0   19.403      5.374   3.611  0.00279 **
AH0513.M8 - AH0212.F1 == 0   10.071      5.708   1.764  0.39077
AH0212.M2 - AH0212.M1 == 0   23.719      5.348   4.435  < 0.001 ***
AH0513.M8 - AH0212.M1 == 0   14.387      5.682   2.532  0.08218 .
AH0513.M8 - AH0212.M2 == 0   -9.332      5.589  -1.670  0.44877
```

- Interaction between replicate and strain: not considered
- P value adjustment: stepwise procedure by Westfall

```
Linear Hypotheses:
                           Estimate Std. Error z value Pr(>|z|)
AH0212.F1 - HWE == 0        -14.620      4.684  -3.121  0.00503 **
AH0212.M1 - HWE == 0        -18.936      4.651  -4.071  < 0.001 ***
AH0212.M2 - HWE == 0          4.783      4.533   1.055  0.49788
AH0513.M8 - HWE == 0         -4.549      4.926  -0.923  0.58496
AH0212.M1 - AH0212.F1 == 0   -4.316      5.474  -0.788  0.58496
AH0212.M2 - AH0212.F1 == 0   19.403      5.374   3.611  0.00163 **
AH0513.M8 - AH0212.F1 == 0   10.071      5.708   1.764  0.14936
AH0212.M2 - AH0212.M1 == 0   23.719      5.348   4.435  < 0.001 ***
AH0513.M8 - AH0212.M1 == 0   14.387      5.682   2.532  0.04139 *
AH0513.M8 - AH0212.M2 == 0   -9.332      5.589  -1.670  0.28943
```

Considering the interaction between replicate and line did not change the result.

2) Fertility with yeast

- Interaction between replicate and strain: not considered
- P value adjustment: single-step method

```
Linear Hypotheses:
                             Estimate Std. Error z value Pr(>|z|)
AH0212.F1 - HWE == 0          -0.2595     0.1141  -2.275   0.1515
AH0212.M1 - HWE == 0          -0.3069     0.1124  -2.730   0.0491 *
AH0212.M2 - HWE == 0          -0.8546     0.1141  -7.492   <0.001 ***
AH0513.M8 - HWE == 0          -0.5332     0.1132  -4.710   <0.001 ***
AH0212.M1 - AH0212.F1 == 0  -0.0474     0.1310  -0.362   0.9963
AH0212.M2 - AH0212.F1 == 0  -0.5951     0.1325  -4.493   <0.001 ***
AH0513.M8 - AH0212.F1 == 0  -0.2737     0.1317  -2.078   0.2272
AH0212.M2 - AH0212.M1 == 0  -0.5477     0.1310  -4.181   <0.001 ***
AH0513.M8 - AH0212.M1 == 0  -0.2263     0.1303  -1.738   0.4077
AH0513.M8 - AH0212.M2 == 0   0.3214     0.1317   2.440   0.1035
```

- Interaction between replicate and strain: not considered
- P value adjustment: stepwise procedure by Westfall

```
Linear Hypotheses:
                             Estimate Std. Error z value Pr(>|z|)
AH0212.F1 - HWE == 0          -0.2595     0.1141  -2.275   0.0453 *
AH0212.M1 - HWE == 0          -0.3069     0.1124  -2.730   0.0235 *
AH0212.M2 - HWE == 0          -0.8546     0.1141  -7.492   <0.001 ***
AH0513.M8 - HWE == 0          -0.5332     0.1132  -4.710   <0.001 ***
AH0212.M1 - AH0212.F1 == 0  -0.0474     0.1310  -0.362   0.7175
AH0212.M2 - AH0212.F1 == 0  -0.5951     0.1325  -4.493   <0.001 ***
AH0513.M8 - AH0212.F1 == 0  -0.2737     0.1317  -2.078   0.0943 .
AH0212.M2 - AH0212.M1 == 0  -0.5477     0.1310  -4.181   <0.001 ***
AH0513.M8 - AH0212.M1 == 0  -0.2263     0.1303  -1.738   0.0943 .
AH0513.M8 - AH0212.M2 == 0   0.3214     0.1317   2.440   0.0292 *
```

- Interaction between replicate and strain: considered
- P value adjustment: single-step method

```
Linear Hypotheses:
                             Estimate Std. Error z value Pr(>|z|)
AH0212.F1 - HWE == 0         -0.26173    0.18661  -1.403   0.6257
AH0212.M1 - HWE == 0         -0.30742    0.18566  -1.656   0.4613
AH0212.M2 - HWE == 0         -0.85642    0.18661  -4.589   <0.001 ***
AH0513.M8 - HWE == 0         -0.53543    0.18611  -2.877   0.0327 *
AH0212.M1 - AH0212.F1 == 0 -0.04569    0.19668  -0.232   0.9994
AH0212.M2 - AH0212.F1 == 0 -0.59469    0.19758  -3.010   0.0220 *
```

```
AH0513.M8 - AH0212.F1 == 0 -0.27371    0.19711  -1.389   0.6347
AH0212.M2 - AH0212.M1 == 0 -0.54900    0.19668  -2.791   0.0417 *
AH0513.M8 - AH0212.M1 == 0 -0.22801    0.19621  -1.162   0.7727
AH0513.M8 - AH0212.M2 == 0  0.32098    0.19711   1.628   0.4787
```

- Interaction between replicate and strain: considered
- P value adjustment: stepwise procedure by Westfall

```
Linear Hypotheses:
                           Estimate Std. Error z value Pr(>|z|)
AH0212.F1 - HWE == 0       -0.26173    0.18661  -1.403   0.2983
AH0212.M1 - HWE == 0       -0.30742    0.18566  -1.656   0.2983
AH0212.M2 - HWE == 0       -0.85642    0.18661  -4.589   <0.001 ***
AH0513.M8 - HWE == 0       -0.53543    0.18611  -2.877   0.0206 *
AH0212.M1 - AH0212.F1 == 0 -0.04569    0.19668  -0.232   0.8163
AH0212.M2 - AH0212.F1 == 0 -0.59469    0.19758  -3.010   0.0140 *
AH0513.M8 - AH0212.F1 == 0 -0.27371    0.19711  -1.389   0.3468
AH0212.M2 - AH0212.M1 == 0 -0.54900    0.19668  -2.791   0.0206 *
AH0513.M8 - AH0212.M1 == 0 -0.22801    0.19621  -1.162   0.3468
AH0513.M8 - AH0212.M2 == 0  0.32098    0.19711   1.628   0.2983
```

3) Fertility with fish food

- Interaction between replicate and strain: not considered
- P value adjustment: single-step method

```
Linear Hypotheses:
                           Estimate Std. Error z value Pr(>|z|)
AH0212.F1 - HWE == 0       -0.03026    0.09835  -0.308  0.99803
AH0212.M1 - HWE == 0       -0.13815    0.09835  -1.405  0.62173
AH0212.M2 - HWE == 0       -0.10405    0.09988  -1.042  0.83419
AH0513.M8 - HWE == 0       -0.35079    0.09835  -3.567  0.00322 **
AH0212.M1 - AH0212.F1 == 0 -0.10789    0.11377  -0.948  0.87650
AH0212.M2 - AH0212.F1 == 0 -0.07379    0.11510  -0.641  0.96790
AH0513.M8 - AH0212.F1 == 0 -0.32053    0.11377  -2.817  0.03850 *
AH0212.M2 - AH0212.M1 == 0  0.03410    0.11510   0.296  0.99830
AH0513.M8 - AH0212.M1 == 0 -0.21264    0.11377  -1.869  0.33127
AH0513.M8 - AH0212.M2 == 0 -0.24674    0.11510  -2.144  0.19947
```

- Interaction between replicate and strain: not considered
- P value adjustment: step-wise procedure by Westfall

```
Linear Hypotheses:
                           Estimate Std. Error z value Pr(>|z|)
AH0212.F1 - HWE == 0       -0.03026    0.09835  -0.308  0.94159
AH0212.M1 - HWE == 0       -0.13815    0.09835  -1.405  0.49358
AH0212.M2 - HWE == 0       -0.10405    0.09988  -1.042  0.54862
AH0513.M8 - HWE == 0       -0.35079    0.09835  -3.567  0.00329 **
AH0212.M1 - AH0212.F1 == 0 -0.10789    0.11377  -0.948  0.60962
```

```
AH0212.M2 - AH0212.F1 == 0 -0.07379    0.11510  -0.641  0.60962
AH0513.M8 - AH0212.F1 == 0 -0.32053    0.11377  -2.817  0.02524 *
AH0212.M2 - AH0212.M1 == 0  0.03410    0.11510   0.296  0.94159
AH0513.M8 - AH0212.M1 == 0 -0.21264    0.11377  -1.869  0.19912
AH0513.M8 - AH0212.M2 == 0 -0.24674    0.11510  -2.144  0.11066
```

- Interaction between replicate and strain: considered
- P value adjustment: single-step method

```
Linear Hypotheses:
                           Estimate Std. Error z value Pr(>|z|)
AH0212.F1 - HWE == 0       -0.03100    0.17887  -0.173    1.000
AH0212.M1 - HWE == 0       -0.13889    0.17887  -0.776    0.937
AH0212.M2 - HWE == 0       -0.10486    0.17968  -0.584    0.978
AH0513.M8 - HWE == 0       -0.35153    0.17887  -1.965    0.283
AH0212.M1 - AH0212.F1 == 0 -0.10789    0.18694  -0.577    0.978
AH0212.M2 - AH0212.F1 == 0 -0.07386    0.18771  -0.393    0.995
AH0513.M8 - AH0212.F1 == 0 -0.32053    0.18694  -1.715    0.425
AH0212.M2 - AH0212.M1 == 0  0.03403    0.18771   0.181    1.000
AH0513.M8 - AH0212.M1 == 0 -0.21264    0.18694  -1.138    0.786
AH0513.M8 - AH0212.M2 == 0 -0.24667    0.18771  -1.314    0.682
```

- Interaction between replicate and strain: considered
- P value adjustment: step-wise procedure by Westfall

```
Linear Hypotheses:
                           Estimate Std. Error z value Pr(>|z|)
AH0212.F1 - HWE == 0       -0.03100    0.17887  -0.173    0.979
AH0212.M1 - HWE == 0       -0.13889    0.17887  -0.776    0.865
AH0212.M2 - HWE == 0       -0.10486    0.17968  -0.584    0.865
AH0513.M8 - HWE == 0       -0.35153    0.17887  -1.965    0.283
AH0212.M1 - AH0212.F1 == 0 -0.10789    0.18694  -0.577    0.865
AH0212.M2 - AH0212.F1 == 0 -0.07386    0.18771  -0.393    0.865
AH0513.M8 - AH0212.F1 == 0 -0.32053    0.18694  -1.715    0.316
AH0212.M2 - AH0212.M1 == 0  0.03403    0.18771   0.181    0.979
AH0513.M8 - AH0212.M1 == 0 -0.21264    0.18694  -1.138    0.621
AH0513.M8 - AH0212.M2 == 0 -0.24667    0.18771  -1.314    0.503
```

4) larval development

- Interaction between replicate and strain: not considered
- P value adjustment: single-step method

```
Linear Hypotheses:
                            Estimate Std. Error z value Pr(>|z|)
AH0212.F1 - HWE == 0        0.033809   0.003412   9.909   <0.001 ***
AH0212.M1 - HWE == 0        0.087296   0.003403  25.655   <0.001 ***
AH0212.M2 - HWE == 0        0.045345   0.003574  12.686   <0.001 ***
AH0513.M8 - HWE == 0        0.042702   0.003505  12.183   <0.001 ***
```

```
AH0212.M1 - AH0212.F1 == 0  0.053487   0.003945  13.559   <0.001 ***
AH0212.M2 - AH0212.F1 == 0  0.011536   0.004096   2.817   0.0384 *
AH0513.M8 - AH0212.F1 == 0  0.008893   0.004032   2.206   0.1753
AH0212.M2 - AH0212.M1 == 0 -0.041951   0.004089 -10.260   <0.001 ***
AH0513.M8 - AH0212.M1 == 0 -0.044594   0.004023 -11.084   <0.001 ***
AH0513.M8 - AH0212.M2 == 0 -0.002643   0.004174  -0.633   0.9692
```

- Interaction between replicate and strain: not considered
- P value adjustment: step-wise procedure by Westfall

```
Linear Hypotheses:
                            Estimate Std. Error z value Pr(>|z|)
AH0212.F1 - HWE == 0        0.033809   0.003412   9.909   <0.001 ***
AH0212.M1 - HWE == 0        0.087296   0.003403  25.655   <0.001 ***
AH0212.M2 - HWE == 0        0.045345   0.003574  12.686   <0.001 ***
AH0513.M8 - HWE == 0        0.042702   0.003505  12.183   <0.001 ***
AH0212.M1 - AH0212.F1 == 0  0.053487   0.003945  13.559   <0.001 ***
AH0212.M2 - AH0212.F1 == 0  0.011536   0.004096   2.817   0.0134 *
AH0513.M8 - AH0212.F1 == 0  0.008893   0.004032   2.206   0.0274 *
AH0212.M2 - AH0212.M1 == 0 -0.041951   0.004089 -10.260   <0.001 ***
AH0513.M8 - AH0212.M1 == 0 -0.044594   0.004023 -11.084   <0.001 ***
AH0513.M8 - AH0212.M2 == 0 -0.002643   0.004174  -0.633   0.5266
```

- Interaction between replicate and strain: considered
- P value adjustment: single-step method

```
Linear Hypotheses:
                            Estimate Std. Error z value Pr(>|z|)
AH0212.F1 - HWE == 0        0.033088   0.017630   1.877   0.3299
AH0212.M1 - HWE == 0        0.085996   0.017628   4.878   <0.001 ***
AH0212.M2 - HWE == 0        0.048519   0.017676   2.745   0.0476 *
AH0513.M8 - HWE == 0        0.041852   0.017650   2.371   0.1232
AH0212.M1 - AH0212.F1 == 0  0.052907   0.017740   2.982   0.0240 *
AH0212.M2 - AH0212.F1 == 0  0.015431   0.017788   0.868   0.9088
AH0513.M8 - AH0212.F1 == 0  0.008764   0.017762   0.493   0.9880
AH0212.M2 - AH0212.M1 == 0 -0.037476   0.017785  -2.107   0.2169
AH0513.M8 - AH0212.M1 == 0 -0.044143   0.017760  -2.486   0.0938 .
AH0513.M8 - AH0212.M2 == 0 -0.006667   0.017807  -0.374   0.9958
```

- Interaction between replicate and strain: considered
- P value adjustment: step-wise procedure by Westfall

```
Linear Hypotheses:
                            Estimate Std. Error z value Pr(>|z|)
AH0212.F1 - HWE == 0        0.033088   0.017630   1.877   0.1174
AH0212.M1 - HWE == 0        0.085996   0.017628   4.878   <0.001 ***
AH0212.M2 - HWE == 0        0.048519   0.017676   2.745   0.0311 *
AH0513.M8 - HWE == 0        0.041852   0.017650   2.371   0.0635 .
AH0212.M1 - AH0212.F1 == 0  0.052907   0.017740   2.982   0.0153 *
AH0212.M2 - AH0212.F1 == 0  0.015431   0.017788   0.868   0.6608
```

```
AH0513.M8 - AH0212.F1 == 0  0.008764   0.017762   0.493   0.6608
AH0212.M2 - AH0212.M1 == 0 -0.037476   0.017785  -2.107   0.0690 .
AH0513.M8 - AH0212.M1 == 0 -0.044143   0.017760  -2.486   0.0470 *
AH0513.M8 - AH0212.M2 == 0 -0.006667   0.017807  -0.374   0.7081
```

5) Male longevity

- Interaction between replicate and strain: not considered
- P value adjustment: single-step method

```
Linear Hypotheses:
                           Estimate Std. Error z value Pr(>|z|)
AH0212.F1 - HWE == 0        0.04927    0.04302   1.145  0.77996
AH0212.M1 - HWE == 0        0.15974    0.04240   3.767  0.00151 **
AH0212.M2 - HWE == 0       -0.02208    0.04584  -0.482  0.98887
AH0513.M8 - HWE == 0       -0.08014    0.04440  -1.805  0.36743
AH0212.M1 - AH0212.F1 == 0  0.11047    0.04905   2.252  0.15877
AH0212.M2 - AH0212.F1 == 0 -0.07136    0.05216  -1.368  0.64504
AH0513.M8 - AH0212.F1 == 0 -0.12942    0.05080  -2.548  0.07939 .
AH0212.M2 - AH0212.M1 == 0 -0.18183    0.05141  -3.536  0.00361 **
AH0513.M8 - AH0212.M1 == 0 -0.23988    0.05020  -4.778  < 1e-04 ***
AH0513.M8 - AH0212.M2 == 0 -0.05806    0.05289  -1.098  0.80564
```

- Interaction between replicate and strain: not considered
- P value adjustment: step-wise procedure by Westfall

```
Linear Hypotheses:
                           Estimate Std. Error z value Pr(>|z|)
AH0212.F1 - HWE == 0        0.04927    0.04302   1.145  0.44063
AH0212.M1 - HWE == 0        0.15974    0.04240   3.767  < 0.001 ***
AH0212.M2 - HWE == 0       -0.02208    0.04584  -0.482  0.62999
AH0513.M8 - HWE == 0       -0.08014    0.04440  -1.805  0.16646
AH0212.M1 - AH0212.F1 == 0  0.11047    0.04905   2.252  0.08488 .
AH0212.M2 - AH0212.F1 == 0 -0.07136    0.05216  -1.368  0.35545
AH0513.M8 - AH0212.F1 == 0 -0.12942    0.05080  -2.548  0.05185 .
AH0212.M2 - AH0212.M1 == 0 -0.18183    0.05141  -3.536  0.00161 **
AH0513.M8 - AH0212.M1 == 0 -0.23988    0.05020  -4.778  < 0.001 ***
AH0513.M8 - AH0212.M2 == 0 -0.05806    0.05289  -1.098  0.44063
```

- Interaction between replicate and strain: considered
- P value adjustment: single-step method

```
Linear Hypotheses:
                           Estimate Std. Error z value Pr(>|z|)
AH0212.F1 - HWE == 0        0.06625    0.09119   0.726    0.950
AH0212.M1 - HWE == 0        0.14335    0.09039   1.586    0.506
AH0212.M2 - HWE == 0       -0.05172    0.09274  -0.558    0.981
AH0513.M8 - HWE == 0       -0.08850    0.09145  -0.968    0.870
AH0212.M1 - AH0212.F1 == 0  0.07711    0.09421   0.818    0.925
```

```
AH0212.M2 - AH0212.F1 == 0 -0.11797    0.09652  -1.222    0.738
AH0513.M8 - AH0212.F1 == 0 -0.15475    0.09523  -1.625    0.481
AH0212.M2 - AH0212.M1 == 0 -0.19507    0.09571  -2.038    0.247
AH0513.M8 - AH0212.M1 == 0 -0.23185    0.09446  -2.455    0.101
AH0513.M8 - AH0212.M2 == 0 -0.03678    0.09667  -0.380    0.996
```

- Interaction between replicate and strain: considered
- P value adjustment: step-wise procedure by Westfall

```
Linear Hypotheses:
                           Estimate Std. Error z value Pr(>|z|)
AH0212.F1 - HWE == 0        0.06625    0.09119   0.726    0.731
AH0212.M1 - HWE == 0        0.14335    0.09039   1.586    0.364
AH0212.M2 - HWE == 0       -0.05172    0.09274  -0.558    0.731
AH0513.M8 - HWE == 0       -0.08850    0.09145  -0.968    0.731
AH0212.M1 - AH0212.F1 == 0  0.07711    0.09421   0.818    0.731
AH0212.M2 - AH0212.F1 == 0 -0.11797    0.09652  -1.222    0.440
AH0513.M8 - AH0212.F1 == 0 -0.15475    0.09523  -1.625    0.364
AH0212.M2 - AH0212.M1 == 0 -0.19507    0.09571  -2.038    0.174
AH0513.M8 - AH0212.M1 == 0 -0.23185    0.09446  -2.455    0.101
AH0513.M8 - AH0212.M2 == 0 -0.03678    0.09667  -0.380    0.731
```

6) Female longevity

- Interaction between replicate and strain: considered
- P value adjustment: step-wise procedure by Westfall

```
Linear Hypotheses:
                           Estimate Std. Error z value Pr(>|z|)
AH0212.F1 - HWE == 0         0.4086     1.0363   0.394    0.995
AH0212.M1 - HWE == 0        -7.3111     1.0153  -7.201   <1e-05 ***
AH0212.M2 - HWE == 0        -7.5867     1.1537  -6.576   <1e-05 ***
AH0513.M8 - HWE == 0        -6.7836     1.0872  -6.240   <1e-05 ***
AH0212.M1 - AH0212.F1 == 0  -7.7197     1.1449  -6.743   <1e-05 ***
AH0212.M2 - AH0212.F1 == 0  -7.9952     1.2706  -6.293   <1e-05 ***
AH0513.M8 - AH0212.F1 == 0  -7.1921     1.2095  -5.946   <1e-05 ***
AH0212.M2 - AH0212.M1 == 0  -0.2755     1.2588  -0.219    0.999
AH0513.M8 - AH0212.M1 == 0   0.5275     1.1931   0.442    0.992
AH0513.M8 - AH0212.M2 == 0   0.8031     1.3068   0.615    0.972
```

- Interaction between replicate and strain: not considered
- P value adjustment: step-wise procedure by Westfall

```
Linear Hypotheses:
                           Estimate Std. Error z value Pr(>|z|)
AH0212.F1 - HWE == 0         0.4086     1.0363   0.394    0.913
AH0212.M1 - HWE == 0        -7.3111     1.0153  -7.201   <1e-04 ***
AH0212.M2 - HWE == 0        -7.5867     1.1537  -6.576   <1e-04 ***
AH0513.M8 - HWE == 0        -6.7836     1.0872  -6.240   <1e-04 ***
```

```
AH0212.M1 - AH0212.F1 == 0  -7.7197     1.1449  -6.743   <1e-04 ***
AH0212.M2 - AH0212.F1 == 0  -7.9952     1.2706  -6.293   <1e-04 ***
AH0513.M8 - AH0212.F1 == 0  -7.1921     1.2095  -5.946   <1e-04 ***
AH0212.M2 - AH0212.M1 == 0  -0.2755     1.2588  -0.219    0.913
AH0513.M8 - AH0212.M1 == 0   0.5275     1.1931   0.442    0.913
AH0513.M8 - AH0212.M2 == 0   0.8031     1.3068   0.615    0.913
```

- Interaction between replicate and strain: considered
- P value adjustment: single-step method

```
Linear Hypotheses:
                           Estimate Std. Error z value Pr(>|z|)
AH0212.F1 - HWE == 0         0.4349     1.5680   0.277 0.998701
AH0212.M1 - HWE == 0        -7.1209     1.5579  -4.571  < 1e-04 ***
AH0212.M2 - HWE == 0        -7.9170     1.6633  -4.760  < 1e-04 ***
AH0513.M8 - HWE == 0        -6.9507     1.6037  -4.334 0.000129 ***
AH0212.M1 - AH0212.F1 == 0  -7.5558     1.6474  -4.587  < 1e-04 ***
AH0212.M2 - AH0212.F1 == 0  -8.3519     1.7485  -4.776  < 1e-04 ***
AH0513.M8 - AH0212.F1 == 0  -7.3856     1.6911  -4.367 0.000121 ***
AH0212.M2 - AH0212.M1 == 0  -0.7961     1.7417  -0.457 0.990992
AH0513.M8 - AH0212.M1 == 0   0.1703     1.6821   0.101 0.999976
AH0513.M8 - AH0212.M2 == 0   0.9663     1.7779   0.544 0.982688
```

- Interaction between replicate and strain: considered
- P value adjustment: step-wise procedure by Westfall

```
Linear Hypotheses:
                           Estimate Std. Error z value Pr(>|z|)
AH0212.F1 - HWE == 0         0.4349     1.5680   0.277    0.952
AH0212.M1 - HWE == 0        -7.1209     1.5579  -4.571   <1e-04 ***
AH0212.M2 - HWE == 0        -7.9170     1.6633  -4.760   <1e-04 ***
AH0513.M8 - HWE == 0        -6.9507     1.6037  -4.334   <1e-04 ***
AH0212.M1 - AH0212.F1 == 0  -7.5558     1.6474  -4.587   <1e-04 ***
AH0212.M2 - AH0212.F1 == 0  -8.3519     1.7485  -4.776   <1e-04 ***
AH0513.M8 - AH0212.F1 == 0  -7.3856     1.6911  -4.367   <1e-04 ***
AH0212.M2 - AH0212.M1 == 0  -0.7961     1.7417  -0.457    0.938
AH0513.M8 - AH0212.M1 == 0   0.1703     1.6821   0.101    0.952
AH0513.M8 - AH0212.M2 == 0   0.9663     1.7779   0.544    0.938
```

Supplementary Table S4.

Estimated required and actually investigated sample sizes in the different replicates (rep), always for a power of 80% and a family-wise significance level of 5%

1) fecundity, number of females
Goal: to detect a difference of half of the (unknown) standard deviation between lines

line	required sample size	rep1	rep2	rep3	sum reps
WT	103	72	116	30	218
F1	103	30	53	15	98
M1	103	34	51	15	100
M2	103	32	63	13	108
M8	103	27	43	15	85

2a) fertility yeast, number of eggs
Goal: to detect a difference of at least 10%-points between lines

line	required sample size	rep1	rep2	rep3	sum reps
WT	409	1205	1942	1213	4360
F1	409	556	879	774	2209
M1	409	603	1060	652	2315
M2	409	539	941	689	2169
M8	409	455	932	659	2046

2b) fertility fish food, number of eggs
Goal: to detect a difference of at least 10%-points between lines

line	required sample size	rep1	rep2	rep3	sum reps
WT	409	1995	1737	1441	5173
F1	409	786	796	724	2306
M1	409	856	730	605	2191
M2	409	940	907	741	2588
M8	409	1081	910	652	2643

3) larval development time
Goal: to detect a difference of half of the (unknown) standard deviation between lines

line	required sample size	rep1	rep2	rep3	sum reps
WT	103	682	735	553	1970
F1	103	290	390	282	962
M1	103	288	390	292	970
M2	103	283	374	185	842

M8	103	242	374	275	891

4a) longevity males
Goal: to detect a difference of half of the (unknown) standard deviation between lines

line	required sample size	rep1	rep2	rep3	sum reps
WT	103	146	200	203	549
F1	103	50	115	113	278
M1	103	75	118	96	289
M2	103	74	120	42	236
M8	103	67	115	72	254

4b) longevity females
Goal: to detect a difference of half of the (unknown) standard deviation between lines

line	required sample size	rep1	rep2	rep3	sum reps
WT	103	147	196	191	534
F1	103	76	121	115	312
M1	103	72	118	145	335
M2	103	74	117	40	231
M8	103	68	120	81	269

5) male mating competitiveness
Goal: to detect a difference of at least 10%-points from equal competitiveness, i.e., from 50%

line	required sample size	rep1	rep2	rep3	sum reps
F1	204	48	95	95	238
M1 II	204	38	14	not performed	52
M1 IV	204	94	98	not performed	192
M2	204	40	90	89	219
M8	204	39	78	99	216

Supplementary Table S5.

Primer names, sequences, and melting temperatures (Tm). Primers displayed in a combination of lower case and upper case letters were designed for Gibson cloning. Lowercase letters denote the homologous recombination sequence of the primer, uppercase letters the actual primer sequence for PCR amplification. The Tm for primers 1-16 was calculated with „A plasmid editor" (http://biologylabs.utah.edu/jorgensen/wayned/ape/), for primers 17-40 Geneious Software (Biomatters Limited) was used.

* the complete primer sequence anneals during PCR amplification, therefore Tm is for complete primer sequence

No.	primer name	primer sequence	Tm (°C)
1	DsRed For-3	CCACAACGAGGACTACACC	56
2	AmCyan Rev+3	CACGGTGGACAGGATGTC	56
3	pUB For-3	CGCACTCGAGCATTTGTG	55
4	pUB Rev+3	GCGAAGAACAGCAAGCC	55
5	AmCyan For1	CCGCCTTCCTGATGCTG	56
6	AmCyanFor2	GAAGCCCGTGACCATGC	57
7	pSLRev1	GCCGAATTCGAATGGCC	55
8	pSLRev2	CAGCTGGCACGACAGG	56
9	M13F	GTAAAACGACGGCCAGT	53
10	M13R-pUC	CAGGAAACAGCTATGAC	47
11	5'SPLNK-PB-SEQ	CGACTGAGATGTCCTAAATGC	54
12	3'SPLNK-PB-SEQ	ACGCATGATTATCTTTAAC	46
13	new iPCR 5'PB	CTATAACGACCGCGTGAGTC	56
14	157R	TGACACTTACCGCATTGACA	56
15	new iPCR 3'PB	CATACTAATAATAAATTCAACAAACAATTTATTTATGT	54
16	3'RevNew2	AAACCTCGATATACAGACCGATAAAACAC	59
17	mfs3	TCCCAGTCACGACGTTGT	58.5
18	mfs10	ACGACCGCGTGAGTCAAAATGACG	66.7
19	mfs11	ATCAGTGACACTTACCGCATTGACA	63.4
20	mfs12	CCTCGATATACAGACCGATAAAACAC	60.1
21	mfs13	TGTAAAACGACGGCCAGT	57.5
22	mfs14	AGGAAACAGCTATGACCAT	54.0
23	mfs30	CAGTGACACTTACCGCATTGACAAGCA	66.6
24	mfs31	CGACTGAGATGTCCTAAATGCACAG	62.4
25	mfs34	CGTACGTCACAATATGATTATCTTTCTAGG	60.9
26	mfs45	CTGCGTTCCGGCGACAAG	62.4
27	mfs47	cggcccacgtggccactagtACGGGAAGTATCAGCTCGACCATGG	66.1
28	mfs48	agcgctctgaagttcCTATACCTTTTGAAGAATAGG	56.3 *
29	mfs49	GAACTTCAGAGCGCTTTTGAAG	59.1
30	mfs50	atatatatattttcttgttatagatatcAATTCGACTTGTACTGACGGACA	60.0

31	P_49	GATCCACAAGGCCCTGAAGC	61.7
32	P_52	GTAATGCAGAAGAAGACCATGGGCTGGGAG	69.2
33	P76	ctaaacaatcggggtacCGCTAGAGTCGACGGTACC	59.4
34	P77	CCTCTACAAATGTGGTATGGCTG	59.8
35	P78	GCCATACCACATTTGTAGAGGT	58.8
36	P139	CTTTTATCGAATTCCTGCAGC	56.3
37	P148	cgcttacaatttacgccTTAAGAGTTATAACTTCGTATAATGTATGCTATACG	60.3
38	P186	TTGTCGCCGGAACGCAGCG	67.2
39	P964	CTAGGCGCGCCAAGCGAAGTTCCTATTCTCTAGAAAGTATAGGAACTTCATCTAATTCAATTAGAGACTAATTC	
40	P965	CGTGGGCCGTGCACCGAAGTTCCTATACTATTTGAAGAATAGGAACTTCGATACATTGATGAGTTTGGAC	
41	P977	ACTGAATTCAGAAGTTCCTATTCTCTAGAAAGTATAGGAACTTCATCTAATTCAATTAGAGACTAATTC	
42	P978	CTAGCTAGCAGAAGTTCCTATACTATTTGAAGAATAGGAACTTCGATACATTGATGAGTTTGGAC	
